# Seed Myco-priming improves crop yield and herbivory induced defenses in maize by coordinating antioxidants and Jasmonic acid pathway

**DOI:** 10.1186/s12870-022-03949-3

**Published:** 2022-12-01

**Authors:** Raufa Batool, Muhammad Jawad Umer, Muhammad Zeeshan Shabbir, Yangzhou Wang, Muhammad Afaq Ahmed, Jingfei Guo, Kanglai He, Tiantao Zhang, Shuxiong Bai, Jie Chen, Zhenying Wang

**Affiliations:** 1 State Key Laboratory for Biology of Plant Diseases and Insect Pest, Institute of Plant Protection, Chinese Academy of Agricultural Sciences, Beijing, 100000 China; 2 State Key Laboratory of Cotton Biology, Institute of Cotton Research, Chinese Academy of Agricultural Sciences (ICR, CAAS), Anyang, China; 3 Institute of Plant Protection, Guangdong Academy of Agricultural Sciences, Guangzhou, China; 4 Insect Ecology, Institute of Plant Protection, Jilin Academy of Agricultural Sciences, Changchun, 130000 China; 5 School of Agriculture and Biology, Shanghai Jiao Tong University, Shanghai, 200000 China

**Keywords:** Seed Myco-priming, Entomopathogenic fungi, *Ostrinia furnacalis*, Antioxidant, Jasmonic acid

## Abstract

**Background:**

Seed Myco-priming based on consortium of entomopathogenic fungi is very effective seed treatment against *Ostrinia furnacalis* herbivory. Maize regulates defense responses against herbivory by the production of defense-related enzymatic and non-enzymatic antioxidants, phytohormones, and their corresponding genes. Jasmonic acid (JA) plays a key role in plant-entomopathogenic fungi-herbivore interaction.

**Results:**

To understand how a consortium of the entomopathogenic fungi *Beauveria bassiana* and *Trichoderma asperellum* induce changes in the response of maize to herbivory and increase the crop yield, 2-year field experiment, antioxidant enzymes, leaf transcriptome, and phytohormone were performed. Fungal inoculation enhanced the production of antioxidant enzymes and JA signaling pathway more than the normal herbivory. The comparison between single inoculated, consortium inoculated, and non-inoculated plants resulted in distinct transcriptome profiles representing a considerable difference in expression of antioxidant- and JA- responsive genes identified through Weighted gene co-expression network analysis (WGCNA) and expression analysis, respectively. Seed priming with a consortium of *B. bassiana* and *T. asperellum* significantly enhanced the expression of genes involved in antioxidants production and JA biosynthesis cascade, with the highest expression recorded at 24-h post *O. furnacalis* larval infestation. They reduced the larval nutritional indices and survival up to 87% and enhancing crop yield and gross return up to 82-96% over the year 2018 and 2019.

**Conclusion:**

From our results we suggest that a consortium of *B. bassiana* and *T. asperellum* can be used synergistically against *O. furnacalis* in maize under field condition and can mediate antioxidants- and JA- associated maize defense response by boosting up the expression of their responsive genes, thereby enhancing crop yield.

**Supplementary Information:**

The online version contains supplementary material available at 10.1186/s12870-022-03949-3.

## Introduction

Maize is the world’s most productive food and industrial crop with 900 million tons of annual production. It provides about 30% of food calories to 57% population of the world [1], along with that maize can be processed into a variety of industrial products, including starch, sweeteners, oil, beverages, glue, industrial alcohol, and fuel ethanol. In the last 10 years, the use of maize for fuel production significantly increased [2]. However, like other crops, maize is also vulnerable to regular attacks by insect herbivores throughout the growing period. *Ostrinia furnacalis* commonly known asAsian corn borer (ACB) is the most destructive pest of maize causing an annual yield loss of about 6 to 9 million tons in East and Southeast Asia, especially China [3]. Plants required a sophisticated defense mechanism through microbes. The development of natural resistance helps the plant to resist insects without agrochemicals. The recent research developed is shifting towards the use of bio-based strategies using the plant beneficial microbes to increase plant defense [4].

Seed priming or pre-soaking is nowadays considered one the most promising techniques in enhancing abiotic and biotic stress tolerance, yield, and growth in crop plants. By altering seed vigor and physiological state, the seed priming approach sets up to improve seed germination and plant development ability [1, 5]. Myco-priming is a technique of seed priming using the fungal biocontrol agents or entomopathogenic fungi to increase crop growth and tolerance against herbivores [6]. However, several studies on the use of fungal biocontrol agents against pests and pathogens are available, but their effect on plant defense response and underlying molecular mechanisms are limited [7]. Numerous biological products based on beneficial microbes including *mychorrhizea, Beauveria, Trichoderma, Pseudomonas*, *Bacillus,* etc. are available and extensively used to promote plant growth and yield, to control plant diseases and pests attack [4].

*Beauveria bassiana* and *Trichoderma asperellum* are widely used biocontrol agents against different biotic stresses, which are capable to stimulate the induced systemic resistance of plants against herbivores by modulating the cross talk between jasmonic acid (JA), and salicylic acid (SA) signaling pathway. *B. bassiana* is a renowned entomopathogenic fungi to controls the pest attack in several plant species through an endophytic interaction with plants [7, 8]. Among 377 identified species of *Trichoderma*, 20 to 30 species were avirulent and used for plant growth and development. Some *Trichoderma* species invade the plant tissues and survive as an endophyte to interact with plant molecular defense responses to release the plant recognized microbial compounds. In addition, it also enhances vegetative, root growth, and grain yield [4]. Recent studies showed that *Trichoderma*-treated plant roots, shoots, and leaves are resistant to insect pest (aphids and caterpillars) [9] and Lepidoptera larvae (*O. furnacalis*) [7, 8].

To counter the herbivore attack, plants have an intricate and dynamic defense response which includes the production of morphological structures, metabolites, volatiles, antioxidant compounds, and proteins. Earlier induction of induced response results in the overall fitness of a plant by reducing the herbivore and insect attacks. Plants with higher activities of antioxidant enzymes i.e., superoxide dismutase (SOD), peroxidase (POD), polyphenol oxidase (PPO), proline, and proteases have a better defense as compared to plants with reduced activities of aforementioned enzymes [5]. The induction of antioxidant activities in response to pest and pathogen invasion is an important research area in recent years [10].

Advances in the insect-plant interaction have improved our knowledge related to developments of defensive strategies implemented by the plants versus the herbivory. Though, the basic mechanisms of defense are much less implied. The differential gene expressions under biotic stress alter the quality and quantity of proteins in plants, which act as signal transduction and oxidative defense. Several proteins such as protease inhibitors, Proteases, chitinases, defensin, and amylases of plants could be intake by insects during consumption to remain stable and intact in the midgut of insect and transport to its hemolymph and affect the growth of insects. Plant hormones such as salicylic acid (SA), ethylene (ET), and jasmonic acid (JA) produced during injury act as a downstream plant defense pathway. Among them, the JA pathway was mainly induced by insect feeding [11, 12].

Jasmonate are responsible for mediating multiple aspects related to plant development like; root and flower development, trichome formation, leaf senescence, and most importantly, plant response to various biotic and abiotic stresses [13]. JA biosynthesis initiates with the oxygenation of α-linoleic acid and its isoleucine-conjugated (JA-Ile) form, which is the active form of JA, starts to accumulate in response to injury or herbivory [14]. In stress conditions, the basic helix-loop-helix (bHLH) transcription factor associated genes, MYC2 binds with the JA responsive elements at the promoter region of genes regulated by JA [15]. Lorenzo et al., [16] reported that MYC2 is responsible for regulating the expression of wound-induced genes like Lipoxygenase (LOX). MYC2 is essential for JA-induced tolerance in *Medicago truncatula* against *Helicoverpa armigera* [17].

Recent advances in omics, as well as quantitative biology, offer numerous ways for the identification of gene networks and their underlying regulatory mechanisms within the living systems. The transcriptome analysis and weighted gene co-expression network analysis (WGCNA) is a promising approach to predicting the gene network [18]. Moreover, the correlation of these modules and phenotypic traits is useful to identify key genes present within the gene networks [19]. So far, this method has not been used to identify candidate genes involved in maize defense against *O. furnacalis* invasion in response to the entomopathogenic fungus at silking stage.

The molecular mechanisms behind the plant defense responses against pests by the beneficial micro-organism can be studied at the meta-organism level. These studies will shed light on the coevolutionary forces shaping insect communities on plants and will offer valuable insights for developing novel strategies of pest control that can modulate plant defense responses. In the present investigation, the transcriptome based WGCNA analysis integrated with antioxidant enzymes identified key candidates responsible for induced defense in maize by seed priming of *B. bassiana* and *T. asperellum* against the Asian corn borer *O. furnacalis*.

## Methods

### Fungal isolates and seed priming for field experiment

The fungus, *B. bassiana* OFDH1-5 (ACCC32726) which was obtained from the Jilin Academy of Agricultural Sciences, Gongzhuling, China, and *T. asperellum* GDFS1009 (CGMCC NO. 9512) which was obtained from the School of Agriculture and Biology, Shanghai Jiao Tong University, Shanghai, China, were kept in Potato dextrose agar medium at 27-28 °C. Fungal suspensions of *B. bassiana* OFDH1-5, as well as *T. asperellum* GDFS1009, have been prepared in a concentration of 1 × 10^9^, and a consortium of fungal suspensions was prepared in a concentration of 5.9 × 10^5^ and 8.4 × 10^8^ for the above mentioned fungal strains, respectively, based on our previous in-vitro and in-vivo bioassay investigation [7, 8]. The seeds of maize variety ‘Jingke 968’, which were obtained from the Institute of plant protection, CAAS, Beijing, China, were surface sterilized for 3 minutes in 0.1% (w/v) HgCl_2_ and cleaned three times with distilled water [20]. Seed priming was done by soaking the surface sterilized seeds in conidial suspension and then placing them in a shaker incubator (HZP-250) at 25-27 ^o^C for 12 hours or overnight. A laminar flow hood was used to dry the seeds. Interaction analysis to verify the synergistic effect of two fungal strains was carried out as published in our previous article [8].

### Field site description and experimental design

The field trial was conducted at the Research Station of the Institute of Plant Protection, CAAS in Gongzhuling, Jilin Province of China (43.53 °N, 124.82°E, and 224.9 m above the sea level). The climate of the experimental station experiences a mild-temperate, semi-humid climate with mean temperatures of 18.5, 14.5, and 5.5 °C and mean annual precipitation of 400-800 mm. The dominant soil type at the research stations is black (Vertosols) with high soil productivity. Maize is annually grown as a monocrop in April- May and harvested in September-October. All experimental plots were managed under the conservation of the Jilin Academy of Agricultural Sciences.

The current experiment was performed during the spring maize season on 11th May 2018 and 6th May 2019. Four treatments were applied including BB (*B. bassiana* OFDH1-5), TA (*T. asperellum* GDFS1009), and BT (*B. bassiana* OFDH1-5 + *T. asperellum* GDFS1009 consortium), and IC (Insect control). Seeds without seed priming were used as the control (IC). Seeds of each treatment were sown in three replicates and 50 plants per replicate were planted (total 150/treatment) with 2.5 m spacing between each treatment to hinder communication and 20 healthy plants/ replicate/ treatment (60 plants/ treatment) were used to collect and analyze the data. Trials were kept covered with ventilated netting (100 μm mesh) from the time of sowing till the end of the experiment to prevent insect immigration. Field conditions, as well as management, have been maintained similar to local agricultural practices. The local weather in July and August 2018 and 2019 was characterized by monsoon rain, raining at least 3-4 days every week (monthly precipitate. Approx. 120-180 mm), with a temperature range of 14 to 29 °C and wind speed ranging between 1.21 and 11.34 m/s. The weather data during the crop growing season in 2018 and 2019 was mentioned in Table S1.

### Insect rearing, infestation, and plant sampling

*O. furnacalis* (Asian corn borer) neonates were received from the Institute of plant protection, CAAS, which were captured from the field and reared in the laboratory using an ACB artificial diet [21]. The field experiment was performed during the spring maize season in 2018 and 2019. For maize crop yield assessment 60 neonate larvae were infested on each healthy plant at silking stage representing second-generation infestation on 10th August 2018 and 6th August 2019 and allowed to feed freely. For larval nutrition analysis and biochemical and transcriptome analysis to find key candidates involved in biochemical and jasmonate-associated defenses, 50 3rd instar larvae were artificially infested on healthy leaves of another group of silking stage maize plants, and to prevent the migration they were covered in small cages made of net. The injured leaves around the larval feeding sites have been collected from 20 developmentally similar plants at various time intervals (0, 12, 24, 48, and 72 hours post-larval infestation (PLI)) and then stored in liquid nitrogen until further use. For larval developmental time and survival rate assessment 50 neonate larvae/ replicate per treatment (total 150 larvae/treatment) were applied and the data was collected from 20 plants per treatment per replicate.

### Crop damage and yield assessment

At the harvest time (9th and 4th September in 2018 and 2019, respectively), the corn ears from 20 developmentally similar plant were harvested. The number of tunnels and their length in *O. furnacalis* damaged corn ears were calculated. The harvested maize ears were sun-dried until they reached 14% moisture content, and then kernel weight/maize ears/treatment was also assessed. The price of maize grain for 2018 and 2019 was obtained from the local market. The average kernel yield per treatment was converted into kg/ha. The additional yield over control was resulted by deducing the yield of the control plots from each treatment plot [22, 23]. The following equations were used for cost-benefit analysis for each treatment:

$$Additional\ Yeild\ over\ Control= Yeild\ of\ Control- Yeild\ of\ treatment$$

$$Gross\ Return= Additional\ Yeild\ over\ Control\times Maize\ Grain\ Price$$

### Larval nutritional analysis

The nutritional indices including consumption index (CI), Approximate digestibility (AD), relative growth rate (RGR), the efficiency of conversion of digested food (ECD), the efficiency of ingested food (ECI) in 3rd instar larvae after feeding for 24 hours were calculated according to the standard formulas of JR Parra et al., [24]. The 3rd leaf from the top was chosen for the trial. The leaf from each plant was first traced on a white paper and then 12 hours starved, pre-weight single larvae were placed manually on each leaf and covered by a net clip cage. Fifty larvae per treatment were applied (one larvae/cage) and replicated three times. Twenty developmentally similar larvae were used to collect the data. The cage was moved every 5-7 hours, depending on the degree of the damaged leaf. The frass from each cage was collected every time the cage was transferred. Finally, the remaining leaf area had been scanned and compared with the traced leaf area on the paper to estimate the actual leaf area consumed. The larvae from each cage were collected and fresh mass was measured [25].

$$CI=\left( Leaf\ ingested\right)\div \left( Larval\ mass\ gain\times Number\ of\ days\right)$$

$$AD=\frac{Leaf\ mass\ ingested- Frass\ mass}{Leaf\ mass\ ingested}$$

$$ECD=\left( Larval\ mass\ gain\right)\div \left( Leaf\ mass\ ingested- Frass\ mass\right)$$

$$ECI=\left( Larval\ mass\ gain\div Leaf\ mass\ ingested\right)$$

$$RGR= RCR\times ECI$$

$$RCR= mg\ consumed/\textrm{mg}\ \textrm{gained}/\textrm{day}$$

### Larval survival rate and developmental time assessment

For estimating the larval survival rates and developmental time from the first instar to the pupae stage the same procedure as described in the above section was followed. Neonate 12-hour starved larvae were placed on the leaf surface in a net clip cage and the cage was moved every day to the newer area of leaf until the larvae were died or developed from the first instar to pupae stage. The experiment was repeated three times with 20 larvae (1 in each cage). Larvae were analyzed daily, and developmental time was recorded.

$$Larval\ survival\ rate\ \left(\%\right)=\left( Alive\ larvae- Total\ number\ of\ larvae\ tested\right)\times 100$$

### Measurement of antioxidant enzyme activities, proline, and chlorophyll contents

The determination of Superoxide dismutase (SOD), as well as peroxidase (POD), was performed by homogenizing 0.5 g of leaf sample in 2 ml of 50 mM PBS containing 1% PVP (PH = 7). Samples were grounded into fine paste under an ice bath and centrifuged for 20 minutes at 16000 x g. The crude enzyme solution was extracted by collecting the supernatant and stored at 4 °C until further use. SOD and POD activity was determined by using blue tetrazolium [26] and using guaiacol, respectively [27]. Protease activity was determined by using a Folin phenol reagent. Polyphenol oxidase was estimated by preparing a reaction mixture containing a buffer (25 mM potassium phosphate buffer) with a pH of 6.8, enzyme extract (0.1 mL), as well as pyrogallol A (0.1 M). Proline content was measured by toluene using 0.1 g of leaf material. Chlorophyll content was determined using 80% acetone. The detailed protocol of all these assays is described in our previous article [7].

### RNA-sequencing and the identification of antioxidants associated candidate genes involved in maize defense against *O. furnacalis*

#### Transcriptome profiling and enrichment analysis

The leaves of primed as well as unprimed treatments at different time intervals (12, 24, 48, and 72 hours) after insect feeding were sampled as three biological replicates. Extraction of total RNA was performed via the TIANGEN kit in Beijing, China as per the manufacturer’s instructions. The sequencing libraries were produced using the NEBNext UltraTM RNA Library Prep Kit (NEB, USA) as per the manufacturer’s instructions. Index codes have been added to attribute the sequences of every sample. The quality of the prepared libraries was checked via Agilent Bioanalyzer, 2100 and libraries were sequenced on an Illumina platform (Illumina HiSeq 2000). A total of 585.31 Gb of clean data with 93.27% of the Q30 base percentage and number of reads ranging from 19,031,339 to 39,227,210 were obtained. The maize reference genome (ZmB73_Ref-Gen_v4) was used to map the clean reads via the Hisat2 tools [28]. The FPKM (fragments number per kilobase of exon model per million) mapped fragments of every single gene were calculated depending on gene length as well as the read count. Differential expression analysis has been analyzed using the DESeq2 R packages (1.20.0). Differential expressions in digital gene data were analyzed. To control the false discovery rate, the resulted *p* values were adjusted with Benjamini and Hochberg’s approach [29]. Genes with adjusted *p*-value < 0.05 have been identified as differentially expressed genes (DEG). Statistical enrichment of DEGs in the Kyoto encyclopedia of genes, and genomes (KEGG) pathways, was carried out using KEGG Orthology Based Annotation System (KOBAS) software [30] and highly enriched defense-related pathways in each treatment were identified [31].

#### Weighted gene co-expression network analysis for identifying hub genes linked to maize protection against Ostrinia furnacalis

R-based package (1.20.0) was utilized to perform the weighted gene co-expression network analysis. Mechanism and a function of defense-related candidate genes associated with antioxidants and chlorophyll parameters and their expression in non-primed and primed seeds with *B. bassiana* OFDH1-5 as well as the *T. asperellum* GDFS1009, versus the *O. furnacalis* were further identified. FPKM values of commonly expressed DEGs were used to perform WGCNA. chlorophyll contents and the activities of antioxidant enzymes were used as a phenotypic character. Significantly associated key modules of the phenotype and genes associated with defense were identified. Moreover, the top genes were selected to target the hub genes in each of the significant modules. Gene networks were visualized with the help of Cytoscape (3.4.0). Hub genes in each network were highlighted in red and have more significant associations with the other genes in the specific network [32].

#### Validation through RT-qPCR

The expression patterns of identified hub genes in significant modules were validated with RT-qPCR using Applied Biosystems 7500 Fast Real-Time PCR System (Applied Biosystems, Foster City, CA) SYBR Green (TAKARA Bio Inc., Japan), and Actin (EU585777.1) as a reference gene. The TIANGEN kit (Beijing, China) was used for the extraction of RNA and the synthesis of cDNA was performed by One-Step gDNA Remover as well as cDNA Synthesis Super Mix obtained from the TransGen Biotech Co., Ltd., Beijing, China, according to the user manual. Samples were amplified at 95 °C (15s), followed up by 40 cycles at 60 °C for the 60s and 95 degrees centigrade for the 30s. Fold changes to the gene-expression levels were calculated using the 2^-ΔΔCT^ method [33]. The primers used are shown in Table S2.

### Defense-related phytohormones by ultra-high performance liquid chromatography-quadrupole time-of-flight mass spectrometry (UHPLC-QTOF-MS*)*

Defense-related phytohormones including, JA, its biosynthetic precursor cis-12-oxo-phytodienoic acid (OPDA), and biologically active JA-Ile were extracted. Already preserved leaf samples were ground via pestle and mortar using liquid nitrogen. Fifty mg of grounded tissue sample was transferred to a tube of 1.5 ml volume and 1 ml solution (isopropanol: water: acetic acid (80:19:1, v/v) has been added. All the samples were stirred up to 30s, sonication was performed for 1h at 4 °C, and centrifuged for 10 minutes at 10,621×g. The supernatant was used for UPLC/ ESI-HR-QTOFMS quantitative analysis of plant hormones as described previously by as suggested by Kasote et al., [5].

### Protein-protein network analysis and prediction of putative miRNA targeting maize *MYC2* transcription factor

The amino acid sequences of the Maize *MYC2* gene were used as the query sequences to get a protein-protein network by using a STRING website (https://cn.string-db.org/). The CDS sequence of the Maize *MYC2* gene was used to identify potential miRNAs via the psRNATarget database (https://www.zhaolab.org/psRNATarget/analysis?function=2, accessed on the 21 May 2022) with standard parameters [34]. The Cytoscape (V3.8.2, https://cytoscape.org/download.html, accessed on the 21 May 2022) software has been utilized to generate the interaction network among the detected miRNAs as well as the corresponding maize *MYC2* Transcription factor*.*

### Statistical analysis

The data obtained from larval nutritional, larval survival and plant damage and yield assessment were analyzed by standard analysis of variance in completely randomized design. The data collected from maize biochemical (antioxidant enzyme, proline, and chlorophyll content), phytohormone and larval developmental time assessment were evaluated by two-way ANOVA with factorial arrangement by using Statistix 8.1 software. The significance of treatment means at *p*-value < 0.05 was tested by using Tukey’s test.

## Results

### Crop damage and yield assessment

Fungal inoculation positively affected the plant damage. The damage caused by larval feeding on corn ear was significantly reduced in fungal inoculated plants as compared to control. Overall highest reduction in tunnel number of about 99.4 and 97.35% was observed in BT followed by 77.6 and 80.7% in BB and 58.02 and 63.5% in TA treatment over the year 2018 and 2019 respectively in comparison to control (IC). Similarly, the tunnel length was substantially reduced by fungal inoculation specially consortium (BT), which reduced tunnel length in corn ear up to 99.8 and 97.8% in 2018 and 2019 respectively. Application of BB and TA treatment reduced tunnel numbers up to 95.2 and 87.7% in 2018 and 92.5 and 83.3% in 2019 over control (IC) (Table 1).

**Table 1:** Plant damage and crop yield assessment data over the year 2018 and 2019 in entomopathogenic fungal inoculated maize under *O. furnacalis* attack

Treatments		No. of tunnels/ear	Length of tunnel/ear (cm)	Yield (kg/ha)	Additional yield over control	Gross return/ha (2.5 CNY/kg)
2	**BB**	0.88 ± 0.03^c^	0.32 ± 0.01^c^	12,792.4 ± 0.01^b^	4518.06 ± 0.12^b^	11,294.15 ± 0.05^b^
0	**TA**	1.58 ± 0.05^b^	0.83 ± 0.11^b^	11,204.7 ± 0.21^c^	2930.19 ± 0.20^c^	7325.40. ± 0.03^c^
1	**BT**	0.02 ± 0.02^d^	0.01 ± 0.01^d^	15,085.7 ± 0.13^a^	6811.62 ± 0.21^a^	17,029.05 ± 0.21^a^
8	**IC**	3.75 ± 0.01^a^	6.79 ± 0.02^a^	8274 ± 0.05^d^	–	–
2	**BB**	1.20 ± 0.21^b^	0.58 ± 0.08^c^	13,096.8 ± 0.03^b^	4688.58 ± 0.04^b^	11,721.45 ± 0.21^b^
0	**TA**	1.92 ± 0.05^b^	1.32 ± 0.10^b^	12,389.9 ± 0.12^bc^	3982.40 ± 0.19^bc^	9956 ± 0.09^c^
1	**BT**	0.14 ± 0.01^c^	0.17 ± 0.01^d^	16,524.9 ± 0.21^a^	8117.45 ± 0.02^a^	20,293.62 ± 0.14^a^
9	**IC**	5.24 ± 0.03^a^	7.79 ± 0.01^a^	8407.5 ± 0.09^d^	–	–

*BB B. bassiana*, *TA T. asperellum*, *BT* consortium of *B. bassiana* and *T. asperellum*, *IC* Insect control. Different lower-case letter followed by mean ± standard error indicates significant difference among treatments as checked by Tukey’s test (*p*-value < 0.05)

The additional yield and gross return were calculated to estimate the increase in corn yield and economic return by the treatment of entomopathogenic fungi. The highest additional yield of 6811.62 KG/ha and 8117.45 KG/ha in year 2018 and 2019 respectively was recorded in consortium treatment (BT), which is an increase of 82 – 96% over control. Treatment by BB and TA resulted in additional yield of 4518.06 (52.2%) and 2930.19 (35.4%) respectively in 2018 year and 4688.58 (55.8%) and 3982.40 (47.5%) in 2019 over control. Similarly, the gross return from the consortium treatment (BT) was higher (17,029.05 and 20,293.62 in 2018 and 2019). Whereas the gross return from BB and TA was recorded as 11,294.15 and 7325.47 in 2018 and 11,721.45 and 9956 was recorded in 2019. It was seen that the yield and gross return was further increased in second year (2019) field trial under entomopathogenic fungal inoculation specially by consortium (BT) (Table 1).

### Effect on larval nutritional indices

**Fig. 1:**
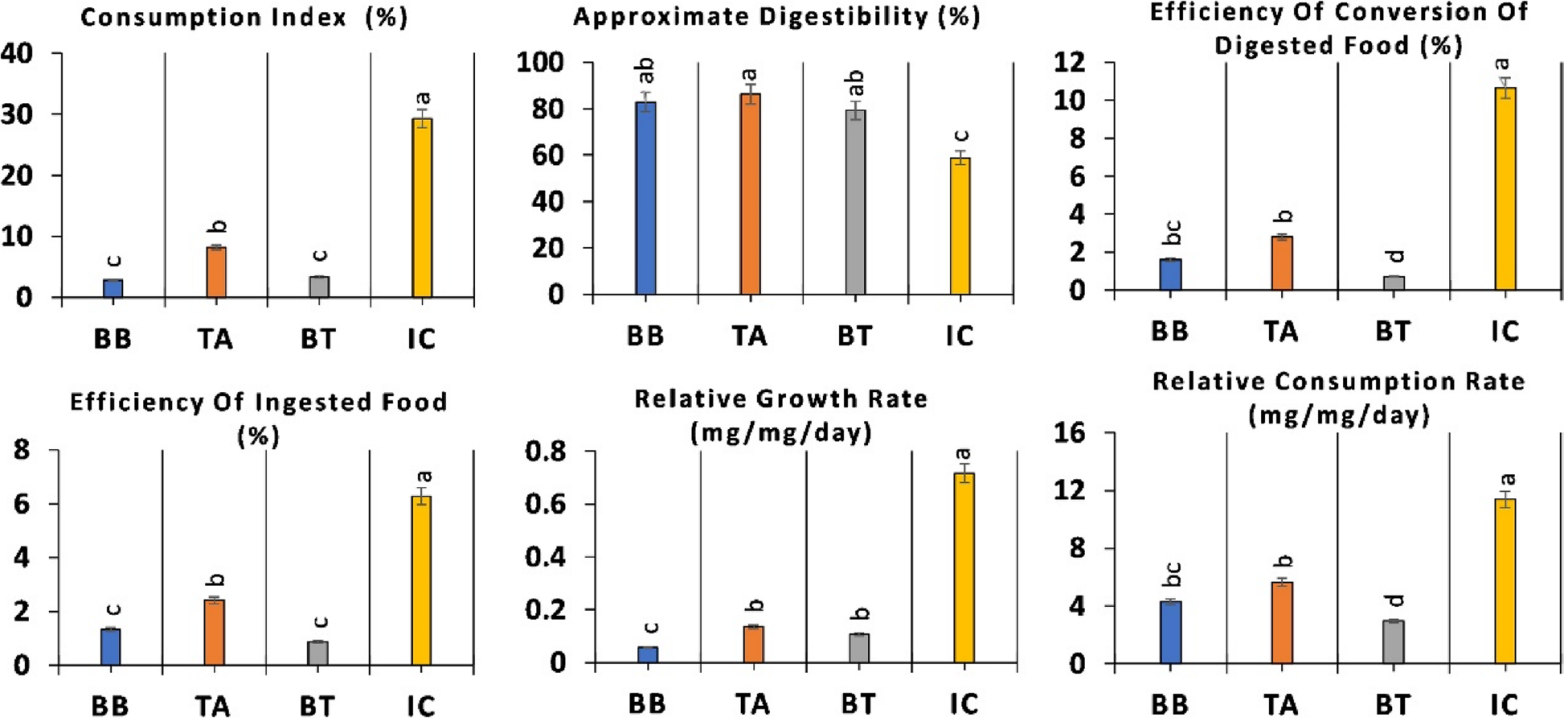
*O. furnacalis* larval nutritional analysis after being fed on *B. bassiana* (BB), *T. asperellum* (TA) and their consortium (BT) inoculated plants in field trial. Different lower-case letter above each bar indicates significant difference among treatments as measured by completely randomized analysis of variance (ANOVA) followed by Tukey’s test (*p-*value < 0.05)

Entomopathogenic fungal inoculation had a great effect on larval performance with significantly reduced relative growth rate (RGR) in comparison with the larvae fed on control plants (Fig. 1). The consumptive index (CI) decreased up to 88% when larvae fed on BB and BT treatment and 72% when larvae fed on TH treated plants in comparison to control. Approximate digestibility (AD), measured to estimate post-ingestive efficiency, was recorded as higher in fungal inoculated plants as compared to control. The larvae fed on control plants ate more, but also produced more frass. In comparison, the efficiency of conversion of digested food (ECD) and efficiency of ingested food (ECI) were reduced up to 93 and 86%, respectively in consortium treatment (BT) followed by 85 and 79% reduction in BB treated plants and 73 and 61% reduction in TH treated plants were recorded (Fig. 1).

### Larval survival rate and developmental time assessment

**Fig. 2:**
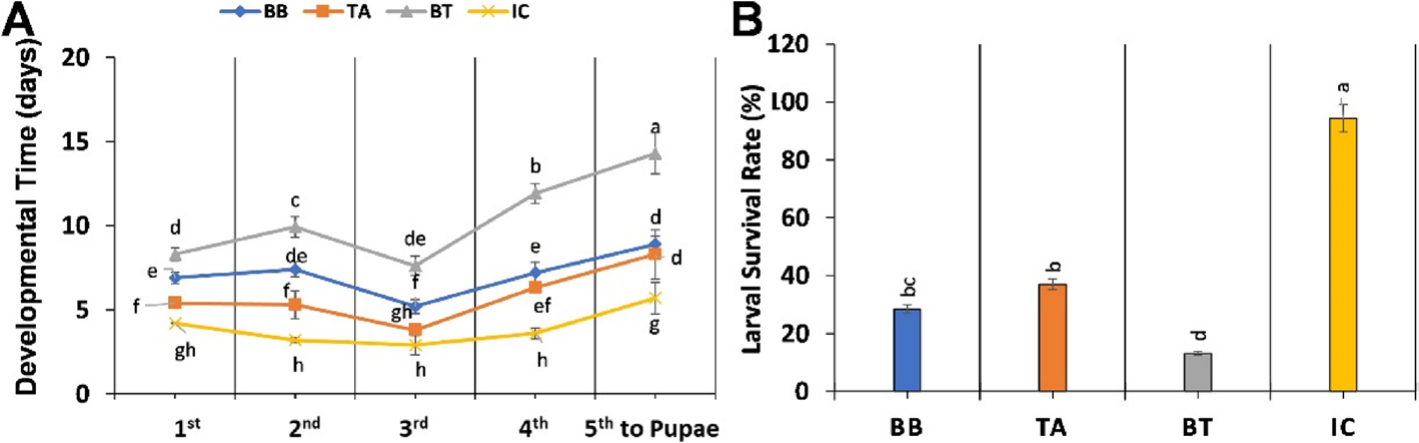
**a** Larval developmental time from 1st instar to pupae stage and **b** Larval survival rate after being fed on B. bassiana (BB), T. asperellum (TA) and their consortium (BT) inoculated plants in field trial. Different lower-case letter above each bar indicates significance difference as measured by analysis of variance two-way ANOVA for ‘A’ and Completely randomized ANOVA for ‘B’ followed by Tukey’s test (*p*-value < 0.05)

The negative effect of maize plant pre inoculated with entomopathogenic fungi was seen in *O. furnacalis* larval development, especially in consortium inoculated plants (BT). Normally a larva takes 3-4 days to develop from one instar to another but the developmental time of larvae from one instar to another was seen to be increased when fed on fungal inoculated plants and a significant delay of 5 to 10 days in larval development was recorded in consortium (BT) treated plants under field condition. A 5th instar larvae take only 4-5 days to pupate whereas larvae fed on BT treated plants took around 12 days to convert into pupae and in BB and TH it took 9 days. Which indicate the slowed growth and developmental process in larvae fed on fungal inoculated plants as compared to control (Fig. 2A).

Similarly larval survival rate was also recorded, and it was seen that more then 50% larvae cannot even reached at 3rd instar stage and died. A significant decrease in larval survival was seen under fungal inoculated plants. The lowest survival rate of 13% was recorded in consortium (BT) treated plants followed by 28% in *B. bassiana* (BB) and 36% in *T. asperellum* (TA) was recorded whereas the survival rate in control group was 94% (Fig. 2B).

### Antioxidant enzyme activity, proline, and chlorophyll content

**Fig. 3:**
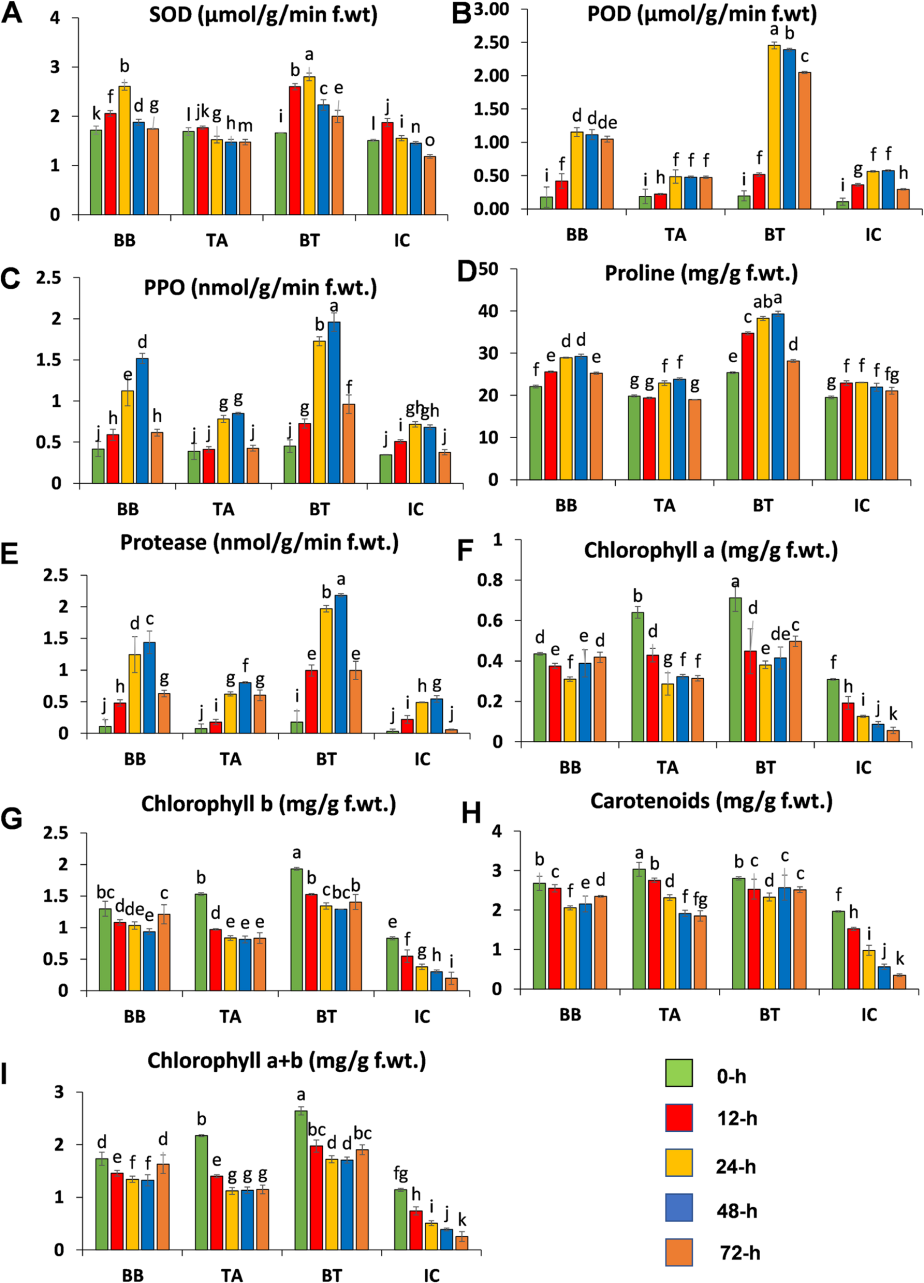
*B. bassiana* OFDH1-5 and *T. asperellum* GDFS1009 induced response of maize antioxidants and chlorophyll content at Silking stage under *O. furnacalis* attack. **a** superoxide dismutase (SOD); **b** peroxidase (POD); **c** polyphenol oxidase (PPO); **d** proline; **e** protease; **f** Chlorophyll a; **g** chlorophyll b; **h** carotenoids; **i** chlorophyll a + b. Error bars indicate standard error and different lower-case letters above each bar indicates significant difference among treatments as measured by analysis of variance two-way ANOVA followed by Tukey’s test (*p-*value < 0.05)

Leaf samples of seed primed and non-primed maize under *O. furnacalis* stress were carefully collected and analyzed for antioxidants, proline, and chlorophyll content at 0-, 12-, 24-, 48- and 72-h PLI. Seed priming with fungal strains significantly increased the enzyme activities at 24-h by the consortium of *B. bassiana* OFDH1-5 and *T. asperellum* GDFS1009 treatment (BT) compared to non-primed control (IC). The seed priming with a consortium of *B. bassiana* OFDH1-5 and *T. asperellum* GDFS1009 (BT) significantly enhanced the SOD, POD, Protease, PPO, and Proline activities up to 80.29-, 336-, 302-, 141-, and 65.8-fold respectively, whereas *B. bassiana* OFDH1-5 (BB) significantly increased SOD, POD, Protease, PPO, and Proline activities up to 67-, 105-, 153-, 65- and 25.4- fold respectively compared to control (IC) at 24-h PLI. *T. asperellum* GDFS1009 inoculation (TA) did not significantly increased the SOD, POD, Protease, PPO, and Proline activities (Fig. 3A-E).

Similarly, chlorophyll content was gradually decreased from 0-h to 48-h of *O. furnacalis* feeding, but fungal inoculations maintained the chlorophyll content, further it increased at 72-h. The chlorophyll-a, chlorophyll-b, and carotenoid contents were increased in BT, followed by BB and TH compared to insect control (IC). Chlorophyll-a, chlorophyll-b, and carotenoids were reduced up to 83-, 88- and 85-folds respectively in the insect control treatment (IC) (Fig. 3F-I).

### RNA-sequencing and the identification of key candidate genes involved in maize defense against *O. furnacalis*

#### Transcriptome profiling

Transcriptome analysis of BB, TA, BT, and IC in three replicates was performed and the comparison efficiency of obtained clean data with reference genome (ZmB73_Ref-Gen_v4), ranged from 78.92 to 88.94% (Table S3). Based on the reference genome comparison, the gene expression level was analyzed and differentially expressed genes were identified with | log2 (fold-change) | > 1 with an adjusted *p*-value < 0.05 for all treatments. Identified genes of all samples were hierarchically clustered relative to control. The colors differentiate the high (red) to the low (green) level of gene expression (Fig. S1A). The principal component analysis revealed the variations between the different samples. Clustering of samples differentiates between each group and control group (IC), which indicates that fungal inoculation especially in consortium treatment (BT) induced changes in gene expression against *O. furnacalis* (Fig. S1B). Eghteen thousand six hundred DEGs were identified in all combinations at different time points from which 10,389 were up-regulated and 8211 were down-regulated (Fig. S1C). From DEGs, the genes involved in metabolic and stress-related pathways, like; Plant hormone signal transduction, Biosynthesis of amino acid, Benzoxazinoid biosynthesis, α-linoleic acid metabolism, plant-stress interaction, and sugar metabolisms were highly enriched based on KEGG enrichment in IC vs BB, IC vs TH, IC vs BT at 12-, 24-, 48-, and 72-h (Fig. S2).

#### Weighted gene co-expression network analysis for the identification of hub genes involved in maize defense against O. furnacalis

**Fig. 4:**
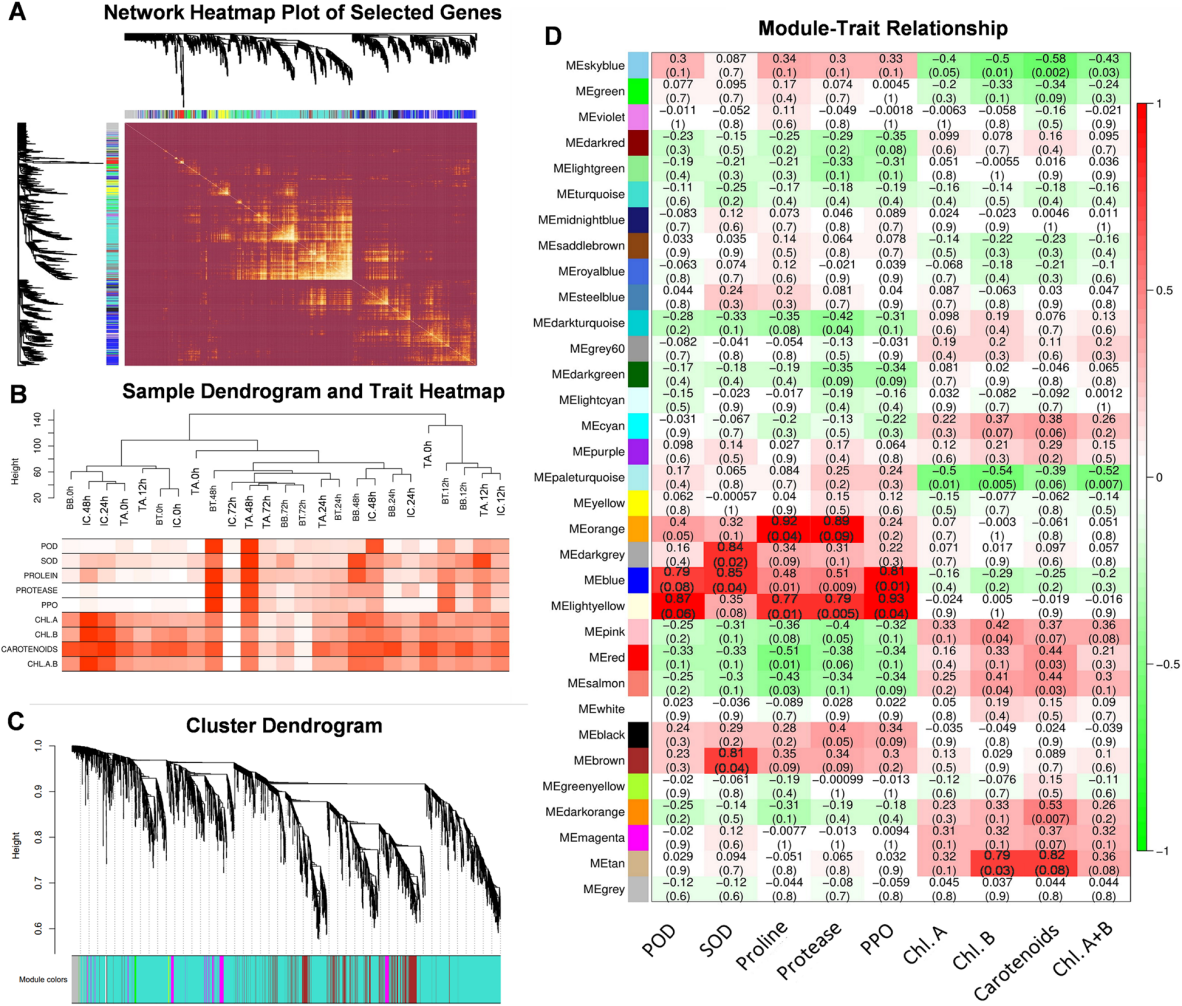
WGCNA analysis to identify key candidate genes involve in maize defense **a** Cluster dendrogram and network heatmap of genes subjected to co-expression module calculation; **b** Sample dendrogram and module trait heatmap of each time point; **c** Hierarchical clustering presenting modules having co-expressed genes. Each leaflet in the tree corresponds to an individual gene; **d** Module-trait associations based on Pearson correlations. The color key from green to red represents *r*^2^ values ranging from − 1 to 1

Co-expression network analysis was performed to identify key candidates involved in the maize defense mechanism. The correlation between the FPKM values of 13,156 common DEGs and contents of antioxidants, proline, and chlorophyll (Fig. 4A) showed that 17 modules were positively correlated. The correlation results were represented with different color codes and presented as cluster dendrogram and network heatmap (Fig. 4B-C). A dendrogram and heat map were constructed to illustrate the expression of each phenotypic trait at 0- 12-, 24-, 48-, and 72-h (Fig. 4D).

**Fig. 5:**
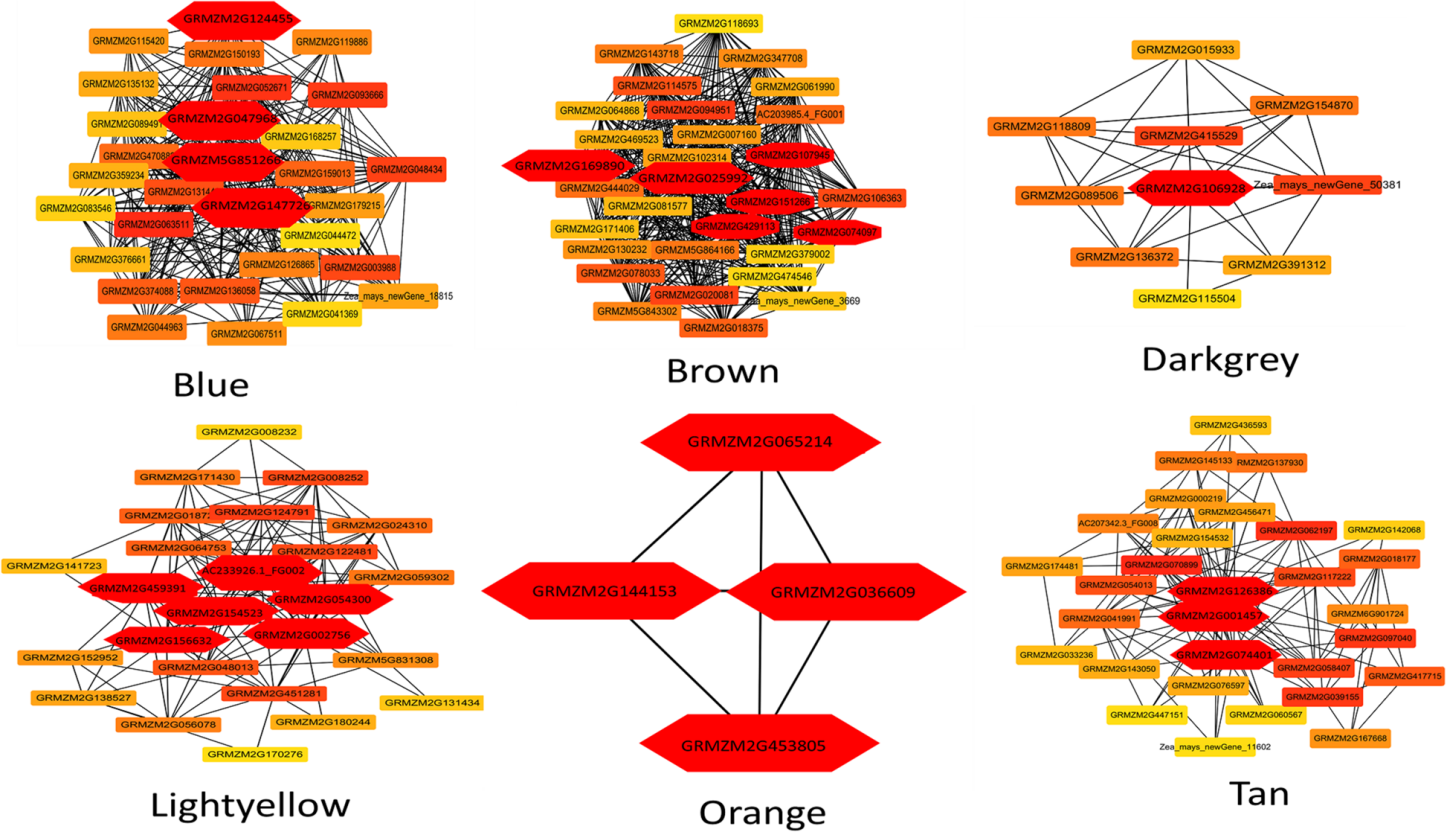
Gene networks of six highly correlated modules with phenotypic traits. Genes in different red shape due to highest weight within module represents hub genes (key candidate genes) and are coded for gene descriptors based on annotation

Six modules were significantly corelated with phenotypic data. The blue module showed significant correlation with SOD (*r*^2^ = 0.85), POD (*r*^2^ = 0.79) and PPO (*r*^2^ = 0.93). Brown and darkgrey modules showed significant correlation with SOD with the correlation coefficient (r^2^) of 0.81 and 0.84. Light yellow module showed positive correlation with POD (*r*^2^ = 0.87), Protease (*r*^2^ = 0.79), PPO (*r*^2^ = 0.81) and proline (*r*^2^ = 0.77). Orange module was significantly corelated with POD (*r*^2^ = 0.89) and Proline (*r*^2^ = 0.92). The tan module has positive correlation with chl. b (*r*^2^ = 0.79) and carotenoids (*r*^2^ = 0.82) (Fig. 4D). The cytoscape built-in extension namely “CytoHubba” was used to select the hub genes from these 6 modules to identify the gene networks (Fig. 5). Twenty-nine hub genes were identified as the major contributor of maize defense response against *O. furnacalis* feeding using gene annotation through the maize reference genome (ZmB73_Ref-Gen_v4).

### RT-qPCR validation of intramodular hub genes

The integrative approach of combining both intramodular hub genes and RT-qPCR validation of hub genes showed the precise identification of key candidate genes. qRT-PCR results were found to be consistent with RNA seq data.

Based on expression analysis, among 29 hub genes, 14 were identified as key candidate genes involved in antioxidants associated maize defense against *O. furnacalis*. Two genes viz. GRMZM2G169890 and GRMZM2G025992 belong to the brown module and the GRMZM2G106928 gene from the dark green module was identified as superoxide dismutase gene ([Cu-Zn] 4AP and [Cu-Zn] 2). In the blue module, GRMZM2G124455, GRMZM2G047968, GRMZM5G851266 genes were identified as superoxide dismutase [Mn] 3.4, L-ascorbate peroxidase 6, and polyphenol oxidase I, respectively. In the lightyellow module, GRMZM2G154523, GRMZM2G054300, GRMZM2G002756, and GRMZM2G156632 genes were identified as Patatin-like protein 1, APx1 - Cytosolic Ascorbate Peroxidase, Clp protease adaptor protein, and wound-induced protease inhibitor, respectively. Three genes viz. GRMZM2G065214, GRMZM2G144153, and GRMZM2G453805 in the orange module were identified as putative Proline-rich extensin-like receptor protein kinase, glutathione peroxidase and chitinase chem 5. GRMZM2G074401 gene in the tan module was identified as omega-3 fatty acid desaturase (Fig. 5).

#### Genes encoding antioxidant enzymes and jasmonate signaling

**Fig. 6:**
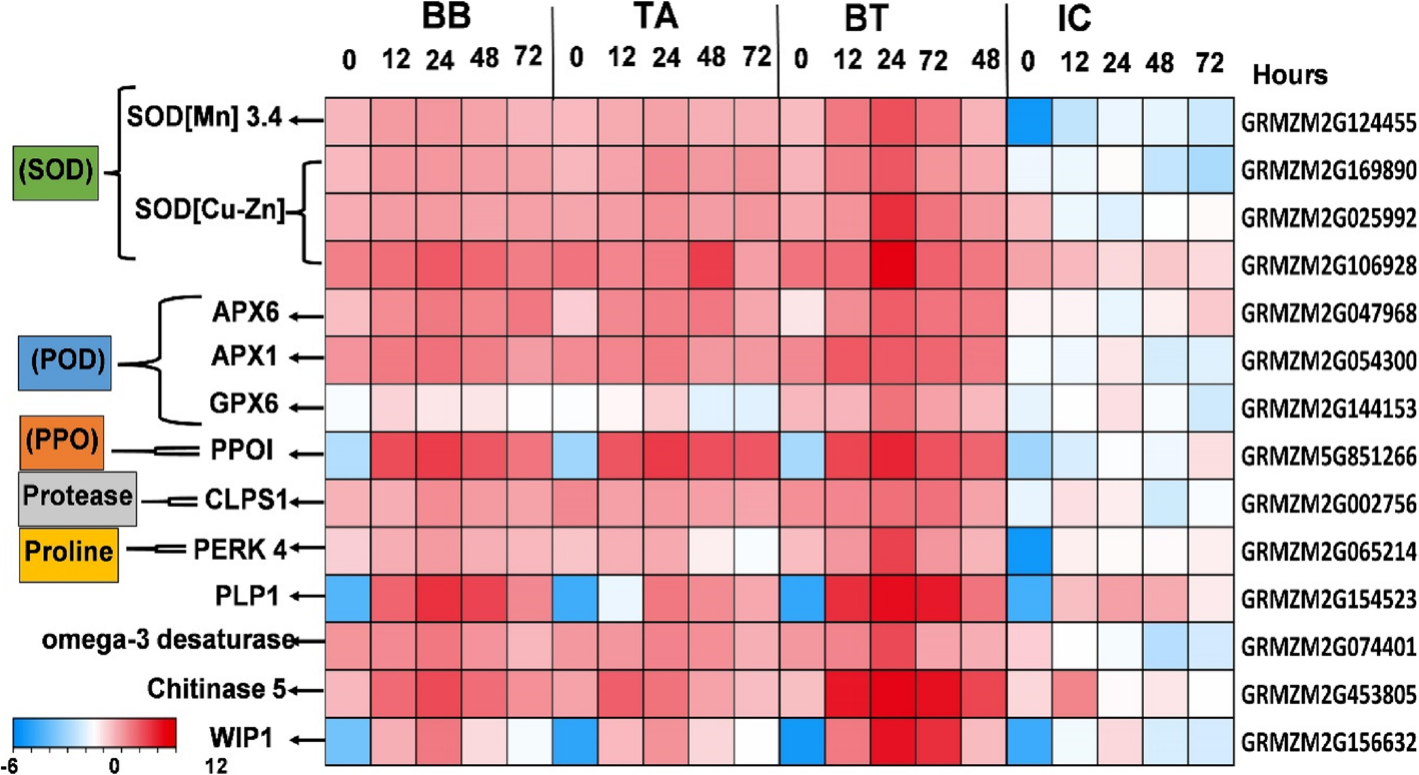
Heat map of log2 fold changes of antioxidants-responsive key genes identified in response to *O. furnacalis* feeding after fungal inoculation. SOD, superoxide dismutase; POD, peroxidase; APX, ascorbate peroxidase; GPX, glutathione peroxidase; PPO, polyphenol oxidase; CLPS1, clp protease1; PERK4, proline-rich receptor-like protein kinase4; PLP1, patatin like protein1; WIP1, wound-induced protein1

All identified key genes were associated with SOD, POD, Protease, proline, and PPO and showed a considerable impact on these traits at 0-, 12-, 24-, 48-, and 72-h PLI. The four genes such as superoxide dismutase Cu, Zn, and Mn, (GRMZM2G124455, GRMZM2G169890, GRMZM2G025992, GRMZM2G106928) are strongly correlated with SOD production, three peroxidase genes (GRMZM2G047968, GRMZM2G054300, and GRMZM2G144153) were strongly associated with POD production, The polyphenol oxidase gene (GRMZM5G851266) was highly correlated with PPO production, The protease associated gene (GRMZM2G002756) was strongly correlated with Protease production and proline-rich extension gene (GRMZM2G065214) was found to be highly correlated with proline content in maize leaves against insect feeding. All these antioxidants associated with gene expression were gradually increased from 12-h to 24-h PLI in seed primed plants. The highest expression was observed in the plants of the seeds primed with the consortium of *B. bassiana* OFDH1-5 and *T. asperellum* GDFS1009 (BT) at 24-h PLI (Fig. 6). The expression patterns of these genes are inconsistent with SOD, POD, PPO, Protease, and proline contents at 0-, 12-, 24-, 48-, and 72-h PLI, respectively.

In the present investigation, we found that two genes viz. GRMZM2G154523 (PLP) and GRMZM2G074401(omega-3 fatty acid desaturase) were strongly associated with jasmonic acid biosynthesis. GRMZM2G453805 (insect cell wall degrading chitinase gene) and insect digestive enzyme inhibitor gene GRMZM2G156632 (Protease inhibitor) are produced in response to the Jasmonic acid synthesis to reduce the feeding of *O. furnacalis*. The expression of these genes was higher in the seeds primed with consortium at 24-h PLI, which was consistent with phenotypic traits (Fig. 6).

### Defense-related phytohormones

**Fig. 7:**
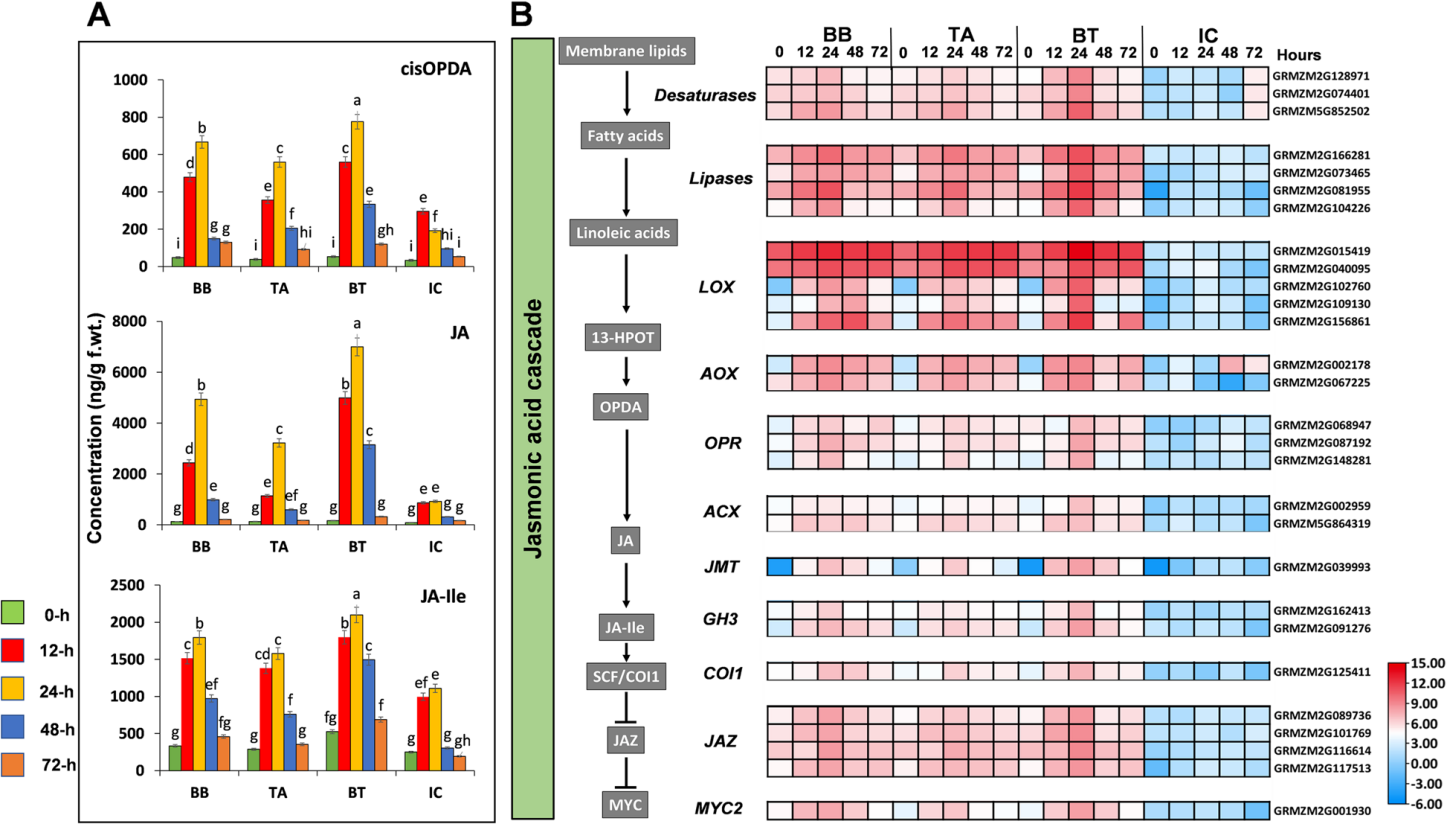
**a** Effect of fungal inoculation in concentration of stress related phytohormone, JA in maize plants. Error bars indicates standard error and different lower-case letters above each bar indicates significant difference among treatments as measured by two-way ANOVA followed by Tukey’s test (*p*-value< 0.05). **b** Heat map of log2 fold changes of genes involved in Jasmonic acid (JA) biosynthesis and signaling cascade. In schemes of cascades, compounds are shown in bold and enzymes in italics. Cis-OPDA, cis-(+)-12-oxo-phytodienoic acid; JA-Ile, JA-isoleucine conjugate; LOX, lipoxygenase; AOS, allene oxide synthase; OPR, oxidoreductase; ACX, acyl coenzyme-A oxidase; GH3, GH3 family proteins; COI1, coronatine-insensitive protein 1; JAZ, Jasmonate ZIM domain; MYC2, bHLH transcription factor

*O. furnacalis* infestation significantly increased *cis*-OPDA, JA, and JA-Ile in all fungal inoculated treatments (BB, TA, BT) but the highest phytohormone content was observed in consortium treated plants (BT), suggesting that the entomopathogenic fungal inoculation., especially consortium (BT) induced the enhanced production of JA metabolites in maize. In contrast, consistently decreases *cis*-OPDA, JA, and JA-Ile content were seen in uninoculated insect control plants (IC). The basal concentration of *cis*-OPDA, JA, and JA-Ile were 75.3-, 86.8-, and 47.1-folds higher in BT at 24-h PLI than IC, respectively, revealing that seed priming with the consortium of *B. bassiana* OFDH1-5 and *T. asperellum* GDFS1009 (BT) can genetically alter the biosynthesis of JA (Fig. 7A).

To further zoom in that how fungal inoculation influenced or boosted the expression of genes involved in JA biosynthesis and signaling cascades, we investigated the differential expression of various genes partaking in the JA biosynthesis pathway influenced by entomopathogenic fungal inoculation (Fig. 7B). Multiple *Desaturases* and *lipase*s genes, that perform a vital role in breaking down the membrane lipids during the formation of alpha-linoleic acid, an intermediate in the biosynthesis of JA were differentially expressed. Some Lipoxygenase (*LOX*) encoding genes, that is responsible for the conversion of linoleic acid to 13(S)- hydroperoxylinolenic (13-*HPOT*), multiple genes encoding Allene oxide synthetase (*AOS*), Oxidoreductase (*OPR*), acyl coenzyme-A oxidase (*ACX*), GH3 family protein (*GH3*), coronatine-insensitive protein 1 (*Col1*), jasmonate ZIM domain (*JAZ*) and bHLH transcription factor (*MYC2*) were identified in JA biosynthesis pathway with differential expression among different treatments (Fig. 7B).

Interestingly, the application of entomopathogenic fungi resulted in significant upregulation of multiple genes upstream of the JA biosynthesis pathway including some candidate genes encoding *desaturases, lipases, LOX, AOS, 12-Oxophytodienoate reductase, Acyl coenzyme-A oxidase.* Also, the transcripts of Jasmonate ZIM domain (*JAZ*) and *MYC2* were induced high expression upon fungal inoculation and the highest expression was observed in the consortium (BT) inoculated plants, especially at 24-h PLI. While in response to *O. furnacalis* in non-inoculated plants (IC) minor changes in the expression of these genes were seen. The higher expression of these genes in BT treated plants indicates that inoculation of plants with a consortium of *B. bassiana* OFDH1-5 and *T. asperellum* GDFS1009 (BT) through seed priming can boost the JA signaling pathway and help plants to better cope with increased defense responses against *O. furnacalis* herbivory in maize, in which *MYC2* transcription factor plays a vital role. (Fig. 7B).

### Protein-protein network analysis and prediction of putative miRNA targeting maize *MYC2* transcription factor

**Fig. 8:**
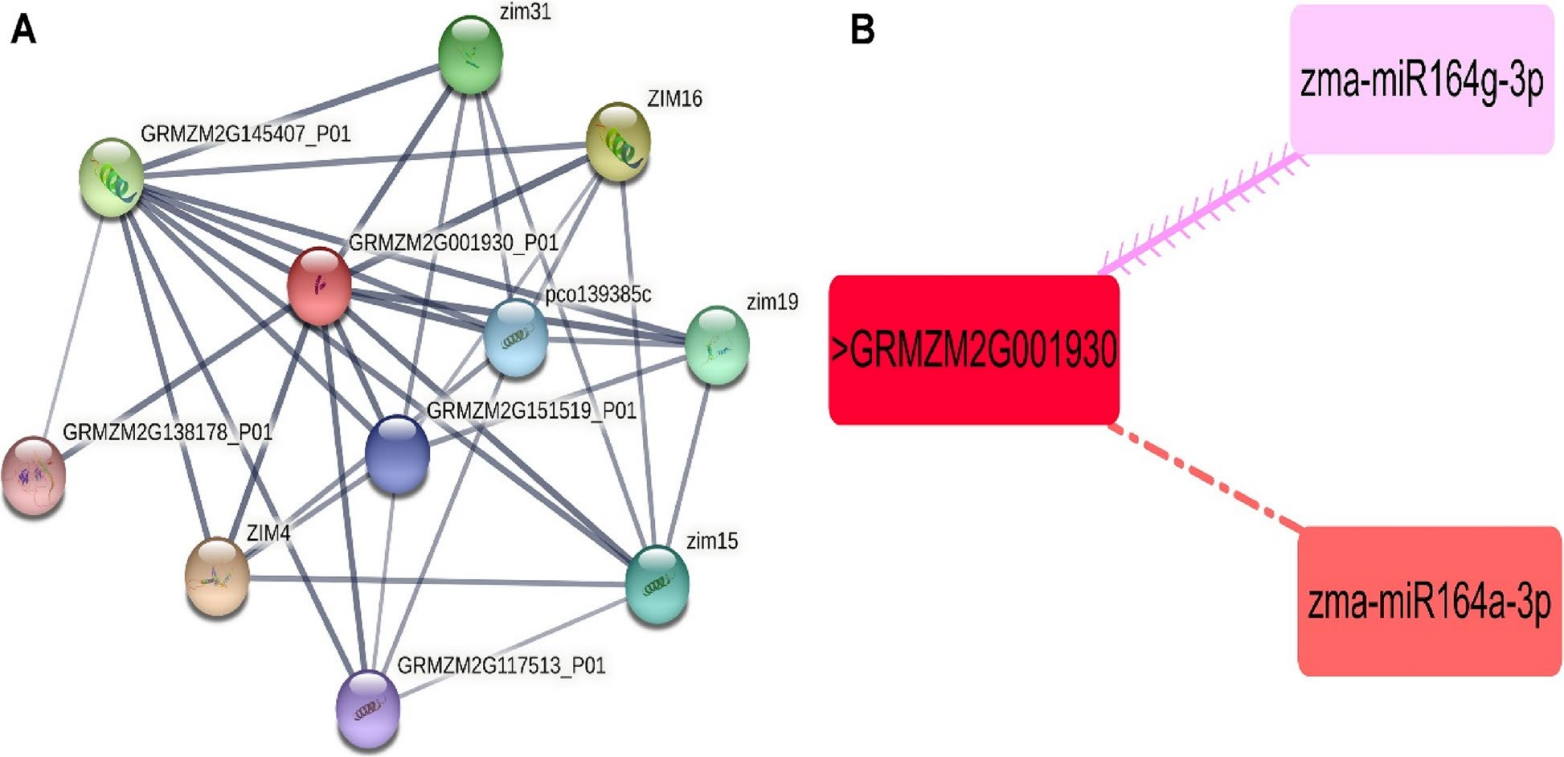
**a** Protein-protein analysis of *MYC2* (GRMZM2G001930) in maize. Colored nodes indicate query proteins and first shell of interactors. **b** A network representation of the regulatory connections among the predicted miRNAs and maize *MYC2* gene. Different colors highlight the interacting miRNAs

To further identify the potential biological functions of *MYC2* in maize, the protein-protein interaction analysis was performed, and 10 potential interactors were detected. Notably, several Jasmonate hormone and response to stress related proteins directly interacted with *MYC2*, suggested its regulatory role in hormone synthesis and defense response. Moreover, *MYC2* showed highly closed relationship with several hormone response proteins, such as Jasmonic acid signaling pathway regulator, Jasmonate *ZIM* domain containing proteins (*ZIM, ZIM16, ZIM31, ZIM4, ZIM19, ZIM15*) (Fig. 8A).

It has been reported previously that miRNAs dependent regulations have significant impacts on plant growth, regulation, and protection. Thus, to strengthen our understanding of the miRNAs associated with the regulation of maize *MYC2* gene that are involved in the jasmonate associated defenses, we identified two putative miRNAs targeting *MYC2* transcription factor (Fig. 8B). The detailed information of the miRNA targeted sites is presented in Table S4. We found that zma-miR164g-3p and zma-miR164a-3p interacts with *MYC2* transcription factor.

## Discussion

High-quality seeds are important for the crop growth, and field performance [20], However, crops have numerous challenges such as environmental stresses, pathogen and pest attacks during their growth and crop yield [8]. *O. furnacalis* feeding on maize causes the most destructive issues during crop cultivation [7]. Therefore, seed quality improvement is necessary to improve the seed’s germination and growth with high yield and protection against insect feeding through antioxidants and phytohormone production [20]. Seed priming using fungal biocontrol agents has recently developed the research interests to improve the morphogenesis, crop growth, defense, and yield [4, 20]. Seed priming is the most effective method to enhance the defense against pests and pathogens, early crop growth, and better yield [8]. The inoculum required for seed priming was lesser than another method [7, 35]. Moreover, the consortium of two biocontrol agents is effective against pest attacks because one acts as a stress inducer and the other as a pest controller [36]. In the current study plants that are seed primed with a consortium of *B. bassiana,* OFDH1-5 and *T. asperellum* GDFS1009 showed higher efficacy against *O. furnacalis* stress compared to the unprimed seeds. The results indicated that seed priming can induce or boosts the defenses in maize against the pest as suggested by Batool et al., [8], hence in the present investigation consortium of *T. asperellum* and *B. bassiana* against *O. furnacalis* was used in both laboratory and field conditions. In current study we recorded a remarkable decrease in maize ear damage caused by *O. furnacalis* after inoculation with entomopathogenic fungi, and a significant increase in yield and gross return of up to 90% was seen especially due to inoculation of fungi as consortium (BT). The larval survival and developmental time were also seriously affected by fungal inoculation resulting in less herbivory in plant and better crop growth. Entomopathogenic *B. bassiana* and *T. asperellum* has the potential to synergistically colonize the plant endophytically when applied in consortium and can boost the defense response in maize in response to herbivory, hence arresting the growth or killing *O. furnacalis* larva [8]. Plants usually provide a suitable environment to entomopathogenic fungi so they can utilize their insecticidal activity and enzymes to reduce herbivory or kill insect pest and boost crop resistance, development and yield [37].

Plant exposure to pests modulates the production of reactive oxygen species (ROS) and induces the enzymatic and non-enzymatic antioxidant defense of plants [38]. In the present investigation, the production of antioxidants and chlorophyll content at 0-, 12-, 24-, 48- and 72-h PLI in seed primed and non-primed maize plants were estimated, and a significant increase in antioxidant content was recorded in seed primed plants, especially in consortium-primed plants. Similarly, consortium-based seed priming maintained the chlorophyll content of the plants under *O. furnacalis* herbivory. SOD, POD, PPO, Protease, and Proline play an essential role in plants against biotic and abiotic stresses [39]. The entomopathogenic fungal inoculants induced plant defenses by activating the expression of antioxidant encoding genes. The previous report proved that the seed priming under biotic and abiotic stresses induced the defensive antioxidants and photosynthetic pigments compared to non-primed plants [8, 40]. The correlation of antioxidants with transcriptomic data through WGCNA showed that 10 SOD, POD, PPO, Protease, and Proline encoding genes and 4 genes participating in JA signaling pathway are important against *O. furnacalis* herbivory defense. All these genes were significantly up-regulated in primed plants under insect feeding with the highest expression recorded in consortium primed plants at 24-h PLI and then the expression gradually decreases at 48-h and 72-h PLI. The seed priming with the consortium of *B. bassiana* OFDH1-5 and *T. asperellum* GDFS1009 promoted the expression of antioxidant encoded genes against insect herbivory.

Interestingly, the chlorophyll content has differed from the expression of photosynthesis-related genes. Hence, a gene related to photosynthesis was not identified during the defense response. Seed primed plants did not show any phenotypic changes or leaf yellowing during the *O. furnacalis* feeding, but photosynthesis-related genes were down-regulated to reduce the photosynthesis for the self-protection against *O. furnacalis* feeding. Previous studies also showed the reduction of chlorophyll-related gene expression might help the plants to adapt the unfavorable conditions [41]. However, within 3 days of feeding the growth and phenotypic condition such as leaf shrinkage and yellowing was observed in the insect control plants, while the seed primed plants were better and consistent with the chlorophyll index.

Phytohormone signaling pathways regulate plant defenses particularly the Jasmonic acid biosynthesis pathway against insect herbivores [42, 43]. Entomopathogenic fungal inoculation boosts the expression of genes related to phytohormones and thereby maintains the phytohormone content to increase plant growth promotion and defense mechanism [7, 44]. Many studies reported the prominent role of JA in maize defense against herbivory by inducing the production of terpenoids and alkaloids. Here we identified that inoculation by consortium induced distinctive transcriptional changes with enhanced expression as compared to single inoculated and non-inoculated plants upon *O. furnacalis* feeding. Fungal inoculation induced genes enriched in responses to wound and biosynthesis of JA. In different plant species, it has been reported that *COI1*- dependent JA signaling mediates the wound responses. However, several plant species also showed *COI1*- independent induction of genes via OPDA.

In the current study, JA biosynthesis genes were upregulated by fungal inoculation, specifically consortium inoculation in response to *O. furnacalis* feeding, caused the accumulation of JA, *cis*-OPDA and JA-Ile and in turns upregulating the JA responsive genes including; Lipoxygenase (*LOX*), 13(S)- hydroperoxylinolenic (13-*HPOT*), Allene oxide synthetase (*AOS*), Oxidoreductase (*OPR*), acyl coenzyme-A oxidase (*ACX*), GH3 family protein (*GH3*), coronatine-insensitive protein 1 (*Col1*), jasmonate ZIM domain (*JAZ*) and bHLH transcription factor (*MYC2*). The expression level of phytohormone and related genes showed a nice trend with the time which started to increase gradually at 12-h PLI and reaches at the highest expression level at 24-h PLI, then the expression starts to decrease at 48-h and 72-h PLI, indicating that 24-h PLI is the peak time when the activity of defensive enzymes and genes was higher. Together, these findings confirm fungal inoculation can boost the maize defenses but the consortium of both entomopathogenic fungi can induce enhanced JA signaling and biosynthesis in Maize than normal herbivory induced defense. Moreover, the significant increase in JA content of the primed plants indicated that seed priming with the consortium of *B. bassiana* OFDH1-5 and *T. asperellum* GDFS1009 genetically regulated the OPDA biosynthesis and modulated JA biosynthesis. According to previous findings plants with higher OPDA levels showed enhanced tolerance against biotic and abiotic stresses [45] Altogether, the present study inferred that the seed priming might maintain the interplay of JA and OPDA to increase the seed germination, plant growth, and defense responses [5, 46].

Maize *MYC2* is a key regulator of JA signaling and exhibit diverse functions by binding to different gene promotors. The protein-protein interaction analysis revealed potential biological functions of *MYC2,* and 10 potential interactors were detected. Notably, several Jasmonate hormone and response to stress related proteins directly interacted with *MYC2* (Jasmonate ZIM domain containing proteins-ZIM, ZIM16, ZIM31, ZIM4, ZIM19, ZIM15), suggesting its regulatory role in hormone synthesis and defense response. MicroRNAs (miRNAs), that are a group of single-stranded, non-coding micro RNAs, are involved in post-transcriptional gene regulation [46]. Various miRNAs have been identified via genome-wide analysis that are involved in growth and development in cotton [47]. In this study we identified two putative miRNAs (zma-miR164g-3p and zma-miR164a-3p) targeting Jasmonic acid biosynthesis domain, *MYC2* transcription factor. Discussed miRNA in current study are all involve in JA associated maize defense mechanism [48]. These studies suggests that these maize-miRNAs might play potential roles in hormone and antioxidants associated plant defense, growth, and development by modifying the transcript level of the *MYC2* genes in maize.

## Conclusion

In summary, the current study demonstrates a comprehensive comparison of entomopathogenic fungal- and normal herbivory-induced defense responses and effect on yield of maize plant through 2-year (2018 and 2019) field experiment, transcriptomic and Jasmonic acid biosynthesis cascade and provides insight into the entomopathogenic fungal induced expression in antioxidant, photosynthesis, and JA responsive genes. The present investigation proved that seed priming with the consortium of *B. bassiana* OFDH1-5 and *T. asperellum* GDFS1009 significantly augmented the maize tolerance against *O. furnacalis* by regulating antioxidants production and jasmonate pathways and in turns enhanced the maize yield. Our results indicated that seed priming technology with entomopathogenic fungi or biocontrol agents had positive significance on plant defense and growth and it can be widely used to enhance crop Yield and protection. *B. bassiana* OFDH1-5 and *T. asperellum* GDFS1009 have a strong synergistic potential and it can be sustainably used to reduce survival of herbivore by increase antioxidants and jasmonate responsive genes against *O. furnacalis* herbivory in maize. Besides, the present study not only showed the mechanism of plant response against unfavorable biotic stress conditions by seed priming with *B. bassiana* OFDH1-5 and *T. asperellum* GDFS1009, but it also laid a foundation for the application of seed priming in maize production against insect herbivory under field conditions to improve yield and seed quality.

## Supplementary Information

**Additional file 1.**

## Data Availability

The datasets generated and analysed during the current study are available in the NCBI repository, Under the bio project number: PRJNA850519. (https://www.ncbi.nlm.nih.gov/bioproject/?term=PRJNA850519).
