# Supplementary material for: Seed Myco-priming improves crop yield and herbivory induced defenses in maize by coordinating antioxidants and Jasmonic acid pathway
Source: BMC Plant Biol. 2022 Dec 1;22:554. doi: 10.1186/s12870-022-03949-3 (PMC9714066; doi:10.1186/s12870-022-03949-3)
Supplement: Supplementary file 1 — Additional file 1. [file 12870_2022_3949_MOESM1_ESM.docx]

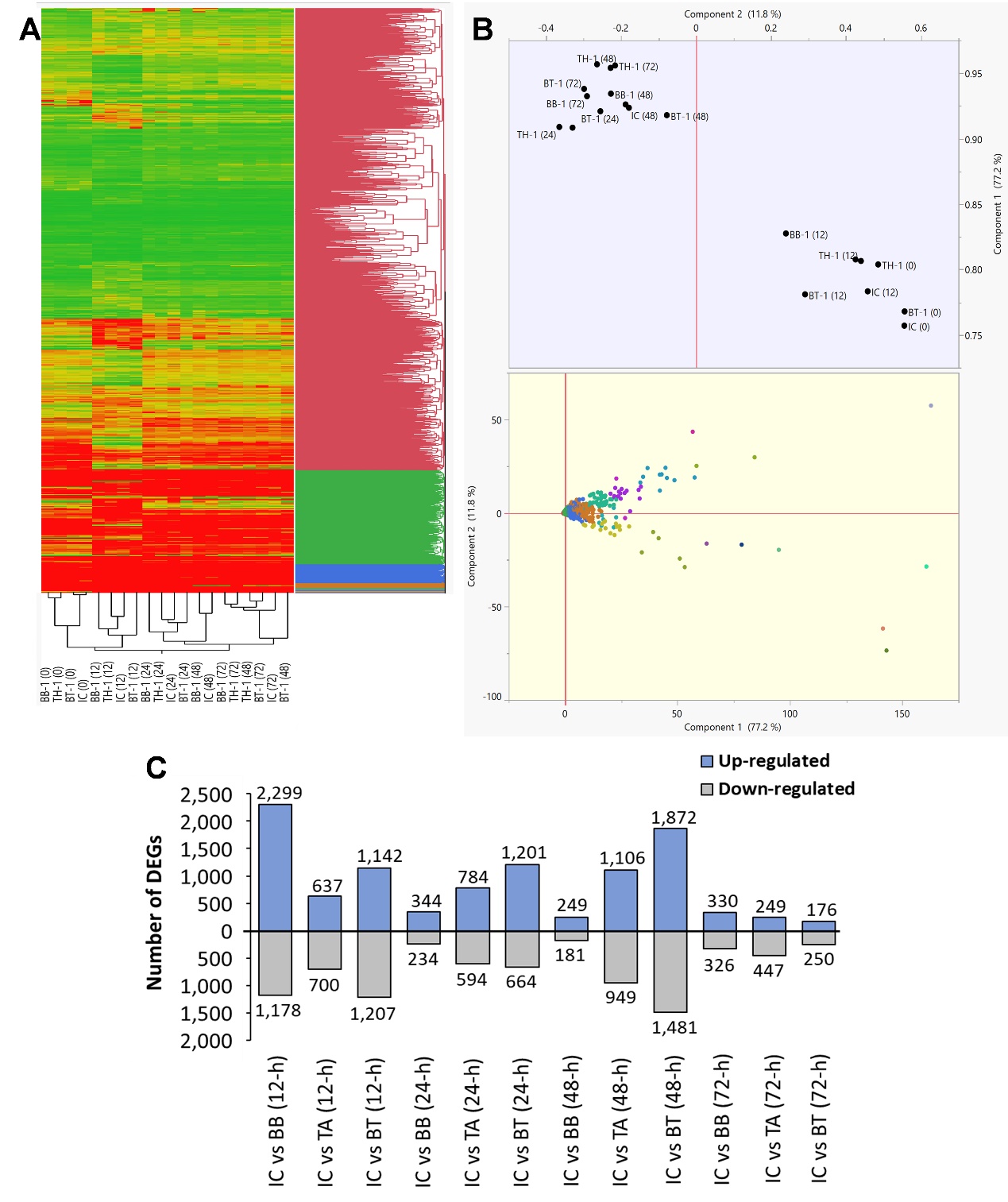


**Figure S1.** Transcriptome response of maize inoculated with single and consortium of *B. bassiana* OFDH1-5 and *T. asperellum* GDFS1009 to *O. furnacalis* (A) Hierarchical Cluster dendrogram of differentially expressed genes, difference in color indicate high (red) and low (green) expression; (B) PCA plot showing variability of transcriptome data. (C) Number of up- and down-regulated differentially expressed genes (DEGs) in each treatment compared to control.


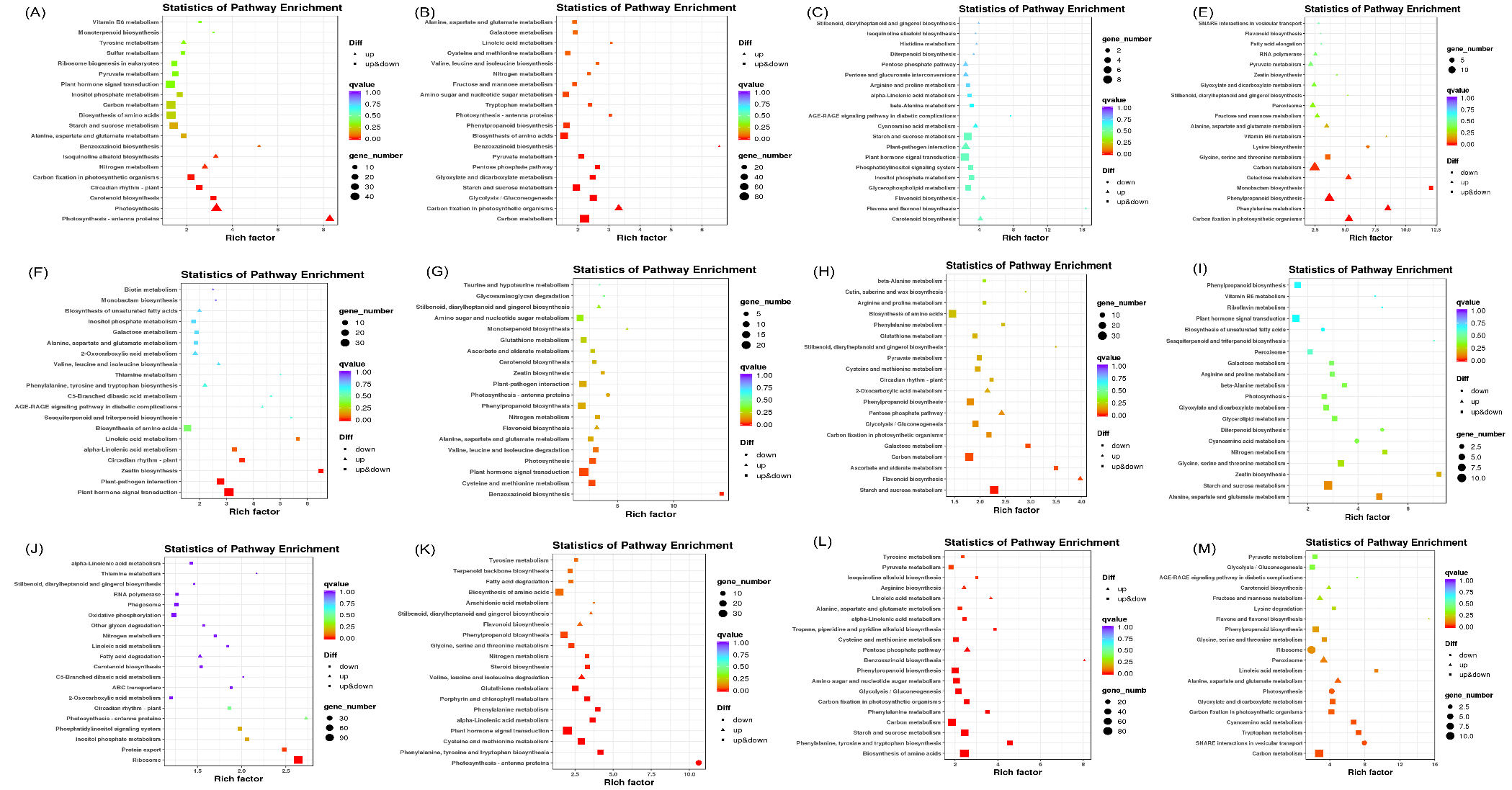


**Figure S2.** KEGG pathway enrichment analysis of DEG's in single and consortium of B. bassiana OFDH1-5 and T. asperellum GDFS1009 inoculated maize leaves induces by O. furnacalis feeding for (A) IC vs BB-1(12-h): (B) IC vs BB-1 (24-h); (C) IC vs BB-1 (48-h): (D) IC vs BB-1 (72-h): (E) IC vs TH-1 (12-h); (F) IC vs TH-1 (24-h); (G) IC vs TH-1 (48-h); (H) IC vs TH-1 (72-h); (I) IC vs BT-1 (12-h); (J) IC vs BT-1 (24-h); (K) IC vs BT-1 (48-h); (L) IC vs BT-1 (72-h) ([www.kegg.jp/kegg/kegg1.html](http://www.kegg.jp/kegg/kegg1.html)).

**Table S1.** Weather data during crop growth season in 2018 and 2019.

| **Date** | **Average temperature (℃)** | | **Average humidity (%)** | | **Wind speed (m/s)** | |
| --- | --- | --- | --- | --- | --- | --- |
|  | **2018** | **2019** | **2018** | **2019** | **2018** | **2019** |
| 6^th^ May | --- | 11.02 | --- | 74.34 | --- | 0.38 |
| 7^th^ May | --- | 10.32 | --- | 78.56 | --- | 0.94 |
| 8^th^ May | --- | 15.63 | --- | 78.15 | --- | 0.20 |
| 9^th^ May | --- | 12.45 | --- | 77.19 | --- | 0.86 |
| 10^th^ May | --- | 13.32 | --- | 81.79 | --- | 0.35 |
| 11^th^ May | 15.34 | 14.43 | 72.73 | 80.64 | 0.85 | 1.02 |
| 12^th^ May | 16.02 | 13.93 | 80.22 | 81.67 | 0.97 | 1.11 |
| 13^th^ May | 14.62 | 14.01 | 81.80 | 81.11 | 1.62 | 1.38 |
| 14^th^ May | 15.44 | 15.00 | 79.39 | 82.83 | 1.98 | 2.34 |
| 15^th^ May | 16.72 | 16.66 | 87.43 | 79.34 | 1.38 | 0.64 |
| 16^th^ May | 17.01 | 16.54 | 81.22 | 81.18 | 0.96 | 0.38 |
| 17^th^ May | 17.22 | 16.20 | 85.03 | 79.25 | 1.66 | 1.34 |
| 18^th^ May | 15.54 | 15.32 | 80.12 | 77.35 | 1.78 | 1.68 |
| 19^th^ May | 15.55 | 16.03 | 75.52 | 75.46 | 1.40 | 2.35 |
| 21^st^ May | 16.03 | 16.93 | 79.45 | 74.66 | 1.69 | 3.24 |
| 22^nd^ May | 17.77 | 16.45 | 80.29 | 78.28 | 1.44 | 1.25 |
| 23^rd^ May | 17.02 | 17.03 | 73.36 | 74.25 | 2.45 | 0.04 |
| 24^th^ May | 17.70 | 17.83 | 74.33 | 77.45 | 1.96 | 0.34 |
| 25^th^ May | 18.32 | 16.99 | 74.67 | 78.34 | 2.18 | 1.29 |
| 26^th^ May | 19.01 | 16.92 | 72.70 | 71.28 | 3.49 | 1.57 |
| 27^th^ May | 19.32 | 17.94 | 72.84 | 79.28 | 3.95 | 2.31 |
| 28^th^ May | 19.92 | 17.99 | 80.95 | 77.31 | 2.68 | 2.94 |
| 29^th^ May | 21.23 | 17.89 | 87.77 | 78.35 | 3.69 | 3.54 |
| 30^th^ May | 18.43 | 21.32 | 84.16 | 81.21 | 4.36 | 1.03 |
| 31^st^ May | 19.05 | 22.92 | 75.26 | 88.23 | 2.38 | 4.31 |
| 1^st^ June | 21.21 | 21.57 | 79.96 | 87.24 | 0.50 | 0.08 |
| 2^nd^ June | 17.72 | 22.91 | 81.33 | 8134 | 1.58 | 2.31 |
| 3^rd^ June | 19.92 | 21.21 | 88.03 | 79.35 | 2.21 | 2.95 |
| 4^th^ June | 18.88 | 20.93 | 78.65 | 77.25 | 3.20 | 2.19 |
| **Date** | **Average temperature (℃)** | | **Average humidity (%)** | | **Wind speed (m/s)** | |
|  | **2018** | **2019** | **2018** | **2019** | **2018** | **2019** |
| 5^th^ June | 18.92 | 24.32 | 74.26 | 89.32 | 1.84 | 2.37 |
| 6^th^ June | 21.20 | 25.69 | 85.37 | 88.24 | 5.40 | 3.31 |
| 7^th^ June | 21.92 | 22.20 | 86.15 | 78.32 | 5.46 | 3.46 |
| 8^th^ June | 22.21 | 25.93 | 86.02 | 75.32 | 4.39 | 5.37 |
| 9^th^ June | 24.42 | 24.76 | 79.18 | 82.14 | 2.41 | 2.41 |
| 10^th^ June | 24.21 | 25.54 | 73.98 | 88.21 | 3.25 | 4.61 |
| 11^th^ June | 24.92 | 25.64 | 82.25 | 84.35 | 3.32 | 5.26 |
| 12^th^ June | 25.62 | 27.43 | 84.39 | 84.31 | 2.21 | 2.38 |
| 13^th^ June | 27.81 | 27.77 | 79.99 | 78.32 | 2.11 | 3.45 |
| 14^th^ June | 29.91 | 27.83 | 78.29 | 82.31 | 2.14 | 3.82 |
| 15^th^ June | 28.86 | 27.34 | 89.94 | 82.31 | 2.54 | 3.16 |
| 16^th^ June | 28.88 | 27.93 | 86.57 | 77.21 | 2.79 | 2.19 |
| 17^th^ June | 29.72 | 28.88 | 81.29 | 73.21 | 0.37 | 2.17 |
| 18^th^ June | 26.82 | 27.63 | 81.53 | 79.56 | 3.14 | 3.54 |
| 19^th^ June | 25.09 | 28.03 | 79.34 | 84.21 | 1.19 | 5.64 |
| 20^th^ June | 25.92 | 27.95 | 85.56 | 88.54 | 1.11 | 2.37 |
| 21^st^ June | 28.02 | 27.02 | 86.34 | 85.34 | 1.28 | 1.34 |
| 22^nd^ June | 27.31 | 27.74 | 86.66 | 79.25 | 2.97 | 3.21 |
| 23^rd^ June | 25.53 | 29.93 | 87.95 | 78.32 | 2.99 | 1.65 |
| 24^th^ June | 25.01 | 29.99 | 86.41 | 72.96 | 3.85 | 3.14 |
| 25^th^ June | 26.03 | 29.83 | 84.67 | 74.47 | 4.97 | 2.31 |
| 26^th^ June | 26.02 | 29.03 | 91.35 | 79.84 | 2.54 | 2.54 |
| 27^th^ June | 26.73 | 28.84 | 95.17 | 76.31 | 1.21 | 2.28 |
| 28^th^ June | 25.54 | 27.98 | 91.34 | 77.28 | 2.54 | 1.34 |
| 29^th^ June | 27.65 | 28.99 | 89.33 | 78.25 | 2.75 | 1.33 |
| 1^st^ July | 29.88 | 29.47 | 87.34 | 75.64 | 1.44 | 2.64 |
| 2^nd^ July | 29.32 | 28.94 | 79.34 | 76.24 | 1.21 | 1.19 |
| 3^rd^ July | 26.73 | 28.83 | 87.21 | 77.54 | 2.61 | 3.56 |
| 4^th^ July | 26.03 | 27.98 | 85.63 | 74.21 | 2.34 | 4.38 |
| 5^th^ July | 26.59 | 27.47 | 86.24 | 71.25 | 1.35 | 4.54 |
| **Date** | **Average temperature (℃)** | | **Average humidity (%)** | | **Wind speed (m/s)** | |
|  | **2018** | **2019** | **2018** | **2019** | **2018** | **2019** |
| 6^th^ July | 27.82 | 28.88 | 88.98 | 75.34 | 1.24 | 4.94 |
| 7^th^ July | 26.93 | 29.97 | 96.75 | 74.48 | 2.64 | 6.42 |
| 8^th^ July | 27.63 | 27.49 | 91.25 | 77.32 | 4.71 | 8.64 |
| 9^th^ July | 28.43 | 27.83 | 89.33 | 79.28 | 1.46 | 9.41 |
| 10^th^ July | 28.01 | 27.53 | 89.25 | 74.74 | 1.54 | 8.31 |
| 11^th^ July | 27.02 | 28.02 | 95.61 | 75.64 | 1.84 | 9.31 |
| 12^th^ July | 26.93 | 28.00 | 94.68 | 89.94 | 1.44 | 1.86 |
| 13^th^ July | 26.77 | 27.90 | 91.34 | 87.56 | 2.97 | 11.34 |
| 14^th^ July | 28.52 | 27.06 | 86.29 | 87.70 | 2.85 | 9.47 |
| 15^th^ July | 27.82 | 28.93 | 91.32 | 81.34 | 2.46 | 5.31 |
| 16^th^ July | 28.84 | 29.03 | 98.34 | 76.31 | 1.94 | 8.32 |
| 17^th^ July | 28.66 | 29.95 | 89.66 | 84.21 | 5.46 | 9.41 |
| 18^th^ July | 27.54 | 29.83 | 86.54 | 88.34 | 7.89 | 6.57 |
| 19^th^ July | 28.02 | 28.65 | 92.74 | 91.34 | 5.61 | 6.28 |
| 20^th^ July | 28.63 | 27.88 | 96.34 | 94.37 | 8.94 | 10.27 |
| 21^st^ July | 27.95 | 28.13 | 89.33 | 89.34 | 4.56 | 11.34 |
| 22^nd^ July | 27.66 | 28.06 | 87.28 | 82.25 | 7.92 | 9.37 |
| 23^rd^ July | 27.07 | 27.79 | 85.29 | 94.31 | 7.10 | 8.51 |
| 24^th^ July | 28.83 | 26.92 | 94.43 | 94.45 | 6.45 | 6.34 |
| 25^th^ July | 28.66 | 27.00 | 91.25 | 96.21 | 4.21 | 8.21 |
| 26^th^ July | 28.73 | 27.51 | 98.34 | 98.84 | 8.23 | 10.31 |
| 27^th^ July | 28.05 | 26.46 | 81.27 | 94.31 | 8.59 | 10.43 |
| 28^th^ July | 29.00 | 26.58 | 98.86 | 88.24 | 8.94 | 11.01 |
| 29^th^ July | 27.94 | 27.82 | 92.22 | 91.47 | 7.32 | 9.41 |
| 1^st^ August | 25.49 | 27.88 | 96.24 | 94.44 | 4.65 | 7.32 |
| 2^nd^ August | 24.43 | 28.64 | 86.38 | 98.21 | 5.44 | 4.31 |
| 3^rd^ August | 24.72 | 28.43 | 89.35 | 91.23 | 4.32 | 4.42 |
| 4^th^ August | 24.77 | 27.46 | 95.64 | 87.21 | 2.34 | 5.34 |
| 5^th^ August | 25.92 | 25.21 | 91.58 | 85.41 | 3.57 | 7.38 |
| 6^th^ August | 23.03 | 22.49 | 91.22 | 79.54 | 6.54 | 8.32 |
| **Date** | **Average temperature (℃)** | | **Average humidity (%)** | | **Wind speed (m/s)** | |
|  | **2018** | **2019** | **2018** | **2019** | **2018** | **2019** |
| 7^th^ August | 19.43 | --- | 98.46 | --- | 4.52 | --- |
| 8^th^ August | 20.92 | --- | 89.35 | --- | 8.41 | --- |
| 9^th^ August | 21.29 | --- | 94.58 | --- | 7.43 | --- |
| 10^th^ August | 24.43 | --- | 96.85 | --- | 4.57 | --- |

**Table S2.** Primer used for qPCR validation of selected key genes.

| **Gene name** | **Forward primer (5′- 3′)** | **Reverse primer (5′- 3′)** |
| --- | --- | --- |
| Actin | TACCATGTTCCCTGGGATTG | GTGGCGCAATCACTTTAACC |
| GRMZM2G169890 | CACACTACAATCCTGCGAGC | GAACAACAACAGCTCTGCCA |
| GRMZM2G025992 | TGCTGACTAGACATGCTGCT | AGAAGATGGTGCCCTTGACA |
| GRMZM2G106928 | TGCATATCGACAGGACCACA | TGGGCCAGTCAAAGGAATCT |
| GRMZM2G124455 | ACCTACGTCGCCAACTACAA | AAATCCTCATCGATGGCCCA |
| GRMZM2G047968 | AACATCTCTATCGCGGCTGA | CATTCATGCCACCTGATCCG |
| GRMZM5G851266 | GCGAAAATGATGGCCTCCTT | CTTGCTTTCCGGCTTGAAGT |
| GRMZM2G154523 | ACCTCTCCGACATCTGCATC | TCCTTGGTGAGCATGGACAT |
| GRMZM2G054300 | GATTTCTACCAGCTTGCCGG | AAAGACTTGCCTCAGGTGGT |
| GRMZM2G002756 | GCTTGATCGGCCAGTAGAGA | TGACAACCCATTCACATGCG |
| GRMZM2G156632 | AAAATGCCCTGGTTGAGAGC | GGTGCACCTGAACTTGTTGT |
| GRMZM2G065214 | ACAAGACCCTCGAGTTCCAC | TGGCTTGGAAGTTGTTGTCG |
| GRMZM2G144153 | TGGCCCAGCTCTATGAGAAG | GAACTTGTAGATGGGCGCAG |
| GRMZM2G453805 | TTCCTCACAACCTTCGGCAA | AGTTGTCCCACAGGTAGTCG |
| GRMZM2G074401 | AGACAAGCTTCCCTGGTACC | AGAGGACCCGACTTCTTTGG |

**Table S3.** Summary of RNA Sequencing data of silking stage maize plant using maize genome as a reference.

| **Samples** | **Clean reads** | **Total mapped** | **GC (%)** | **Q30 (%)** |
| --- | --- | --- | --- | --- |
| BB-1 (0-h) | 23,916,992 | 42,030,503 (87.87%) | 57.22% | 94.58% |
| BB-1 (0-h) | 19,031,339 | 30,892,911 (81.16%) | 56.47% | 94.04% |
| BB-1 (0-h) | 23,347,907 | 38,410,213 (82.26%) | 55.94% | 94.57% |
| BB-1 (12-h) | 32,794,990 | 57,377,978 (87.48%) | 54.71% | 94.63% |
| BB-1 (12-h) | 25,708,135 | 48,546,497 (81.39%) | 54.59% | 94.33% |
| BB-1 (12-h) | 27,233,808 | 47,259,578 (86.77%) | 55.82% | 94.33% |
| BB-1 (24-h) | 23,503,496 | 41,444,082 (88.17%) | 56.22% | 93.35% |
| BB-1 (24-h) | 34,417,768 | 58,516,651 (85.01%) | 54.84% | 94.41% |
| BB-1 (24-h) | 31,946,489 | 54,390,862 (85.13%) | 55.51% | 94.61% |
| BB-1 (48-h) | 21,697,742 | 38,338,462 (88.35%) | 56.06% | 95.17% |
| BB-1 (48-h) | 23,119,311 | 41,073,806 (88.83%) | 56.63% | 95.22% |
| BB-1 (48-h) | 26,623,274 | 47,181,343 (88.61%) | 56.78% | 95.04% |
| BB-1 (72-h) | 24,562,591 | 43,239,904 (88.02%) | 55.34% | 95.28% |
| BB-1 (72-h) | 29,157,005 | 51,303,170 (87.98%) | 55.99% | 95.12% |
| BB-1 (72-h) | 22,544,210 | 39,648,528 (87.94%) | 55.69% | 95.55% |
| TH-1 (0-h) | 30,849,164 | 54,488,259 (88.31%) | 56.05% | 94.67% |
| TH-1 (0-h) | 25,970,459 | 43,716,358 (84.17%) | 55.73% | 94.61% |
| TH-1 (0-h) | 24,173,658 | 38,804,894 (80.26%) | 56.41% | 93.34% |
| TH-1 (12-h) | 26,276,115 | 46,285,519 (88.08%) | 56.77% | 94.83% |
| TH-1 (12-h) | 26,333,713 | 43,142,673 (81.92%) | 55.91% | 94.31% |
| TH-1 (12-h) | 26,840,128 | 46,104,134 (85.89%) | 55.44% | 94.12% |
| TH-1 (24-h) | 39,227,210 | 67,854,588 (86.49%) | 55.60% | 94.50% |
| TH-1 (24-h) | 20,049,198 | 33,460,976 (83.45%) | 56.10% | 94.59% |
| TH-1 (24-h) | 21,643,791 | 36,717,775 (84.82%) | 56.47% | 94.68% |
| TH-1 (48-h) | 28,998,034 | 51,109,795 (88.13%) | 55.31% | 94.53% |
| TH-1 (48-h) | 23,138,162 | 40,925,655 (88.44%) | 56.87% | 95.45% |
| TH-1 (48-h) | 21,839,687 | 38,726,215 (88.66%) | 56.44% | 95.38% |
| TH-1 (72-h) | 21,687,076 | 38,302,391 (88.31%) | 56.16% | 94.78% |
| TH-1 (72-h) | 22,194,707 | 38,441,068 (86.60%) | 56.23% | 95.36% |
| TH-1 (72-h) | 27,948,284 | 47,317,676 (84.65%) | 55.52% | 95.36% |
| BT-1 (0-h) | 29,638,727 | 55,060,030 (88.85%) | 55.23% | 93.89% |
| BT-1 (0-h) | 24,621,991 | 46,966,430 (79.23%) | 55.73% | 94.29% |
| BT-1 (0-h) | 26,979,662 | 39,945,937 (81.12%) | 56.26% | 94.12% |
| BT-1 (12-h) | 23,253,352 | 43,372,033 (80.38%) | 55.32% | 94.45% |
| BT-1 (12-h) | 23,273,785 | 36,773,423 (79.07%) | 55.43% | 93.27% |
| BT-1 (12-h) | 21,601,741 | 38,328,163 (82.34%) | 55.57% | 94.14% |
| **Samples** | **Clean reads** | **Total mapped** | **GC (%)** | **Q30 (%)** |
| BT-1 (24-h) | 19,945,674 | 34,731,826 (87.07%) | 56.67% | 94.85% |
| BT-1 (24-h) | 19,671,446 | 33,700,764 (85.66%) | 56.20% | 94.52% |
| BT-1 (24-h) | 22,813,024 | 39,274,978 (86.08%) | 56.43% | 94.55% |
| BT-1 (48-h) | 23,868,954 | 40,929,117 (85.74%) | 55.09% | 94.81% |
| BT-1 (48-h) | 26,802,968 | 47,536,698 (88.68%) | 56.62% | 94.91% |
| BT-1 (48-h) | 23,454,290 | 40,245,993 (85.80%) | 54.75% | 95.11% |
| BT-1 (72-h) | 23,290,236 | 40,755,804 (87.50%) | 55.00% | 94.53% |
| BT-1 (72-h) | 23,517,922 | 41,460,167 (88.15%) | 55.73% | 95.00% |
| BT-1 (72-h) | 24,005,631 | 42,254,756 (88.01%) | 55.69% | 95.18% |
| IC (0-h) | 32,854,109 | 58,333,111 (88.78%) | 56.23% | 94.26% |
| IC (0-h) | 28,620,472 | 45,334,200 (79.20%) | 55.58% | 94.13% |
| IC (0-h) | 29,873,884 | 47,155,879 (78.92%) | 53.03% | 93.61% |
| IC (12-h) | 28,856,150 | 50,806,860 (88.03%) | 55.63% | 93.62% |
| IC (12-h) | 39,897,035 | 66,618,158 (83.49%) | 55.68% | 94.89% |
| IC (12-h) | 24,418,909 | 40,281,842 (82.48%) | 56.54% | 94.22% |
| IC (24-h) | 22,371,842 | 38,605,107 (86.28%) | 55.96% | 94.61% |
| IC (24-h) | 24,813,363 | 40,239,105 (81.08%) | 55.50% | 94.16% |
| IC (24-h) | 21,700,289 | 37,215,442 (85.75%) | 55.49% | 94.60% |
| IC (48-h) | 19,966,343 | 35,517,692 (88.94%) | 56.51% | 94.56% |
| IC (48-h) | 22,930,428 | 40,700,067 (88.75%) | 55.70% | 95.08% |
| IC (48-h) | 20,296,898 | 36,086,370 (88.90%) | 56.66% | 95.05% |
| IC (72-h) | 21,766,035 | 38,430,300 (88.28%) | 56.28% | 95.29% |
| IC (72-h) | 24,788,252 | 43,765,069 (88.28%) | 56.68% | 94.37% |
| IC (72-h) | 21,208,343 | 37,589,924 (88.62%) | 56.94% | 94.85% |

**Table S4.** The target sites of miRNA interacting with jasmonate associated maize MYC2 transcription factor.

| **miRNA Acc.** | **Target Acc.** | **Expectation** | **UPE$** | **miRNA start** | **miRNA end** | **Target start** | **Target end** | **miRNA aligned fragment** | **Target aligned fragment** | **Inhibition** |
| --- | --- | --- | --- | --- | --- | --- | --- | --- | --- | --- |
| zma-miR164g-3p | >GRMZM2G001930 | 4 | -1 | 1 | 21 | 930 | 950 | CACGUGCUCCCCUUCUCCACC | UUUUGAGAACGGGAGCACGAG | Cleavage |
| zma-miR164a-3p | >GRMZM2G001930 | 5 | -1 | 1 | 21 | 930 | 950 | CACGUGUUCUCCUUCUCCAUC | UUUUGAGAACGGGAGCACGAG | Cleavage |

**Table S5.** Statistical parameters of analysis of variance (ANOVA) for Completely randomized design.

| **Parameters** | **Source** | **(DF)** | **MS** | **F-Value** | ***p*-value** |
| --- | --- | --- | --- | --- | --- |
| No. of tunnels/ear in 2018 | Treatments | 3 | 7.61256 | 1866 | 0.0000 |
|  | Error | 8 | 0.00408 |  |  |
| Length of tunnel/ear in 2018 | Treatments | 3 | 30.6670 | 516 | 0.0000 |
|  | Error | 8 | 0.0594 |  |  |
| Yield in 2018 | Treatments | 3 | 2.45x10^7^ | 3.9x10^9^ | 0.0000 |
|  | Error | 8 | 0.06262 |  |  |
| Additional Yield over control in 2018 | Treatments | 2 | 1.14x10^7^ | 1x10^8^ | 0.0000 |
|  | Error | 6 | 0.09727 |  |  |
| Gross return/ha in 2018 | Treatments | 2 | 7x10^7^ | 1x10^9^ | 0.0000 |
|  | Error | 6 | 0.05017 |  |  |
| No. of tunnels/ear in 2019 | Treatments | 3 | 14.5411 | 262 | 0.0000 |
|  | Error | 8 | 0.0555 |  |  |
| Length of tunnel/ear in 2019 | Treatments | 3 | 38.4414 | 1173 | 0.0000 |
|  | Error | 8 | 0.0328 |  |  |
| Yield in 2019 | Treatments | 3 | 3x10^7^ | 5.3x10^8^ | 0.0000 |
|  | Error | 8 | 0.06224 |  |  |
| Additional Yield over control in 2019 | Treatments | 2 | 1.4x10^7^ | 1.5x10^8^ | 0.0000 |
|  | Error | 6 | 0.09608 |  |  |
| Gross return/ha in 2019 | Treatments | 2 | 9x10^7^ | 1x10^9^ | 0.0000 |
|  | Error | 6 | 0.07750 |  |  |
| Consumption index | Treatments | 3 | 467.666 | 61.8 | 0.00001 |
|  | Error | 8 | 7.566 |  |  |
| Approximate digestibility | Treatments | 3 | 446.630 | 40.6 | 0.00003 |
|  | Error | 8 | 11.000 |  |  |
| Conversion of digested food efficiency | Treatments | 3 | 61.7723 | 16.7 | 0.00084 |
|  | Error | 8 | 3.7078 |  |  |
| Efficiency of ingested food | Treatments | 3 | 6.88847 | 5.97 | 0.01942 |
|  | Error | 8 | 1.15441 |  |  |
| Relative growth rate | Treatments | 3 | 0.28375 | 4.80 | 0.03385 |
|  | Error | 8 | 0.05913 |  |  |
| Relative consumption rate | Treatments | 3 | 41.3216 | 17.7 | 0.0007 |
|  | Error | 8 | 2.3319 |  |  |
| Larval survival rate | Treatments | 3 | 3643.24 | 101 | 0.00000 |
|  | Error | 8 | 36.22 |  |  |

**Table S6.** Statistical parameters of analysis of variance (two-way ANOVA) with factorial design.

|  | **Variable 1 (Treatment)** | **Variable 2**  **(days and Time)** | **Treatment x Time** | **Error** |
| --- | --- | --- | --- | --- |
| Degree of freedom (df) | 3 | 4 | 12 | 40 |
|  | **Source** | **MS** | **F-value** | ***p*-value** |
| Larval developmental time | Treatment x days | 2.522 | 2.08 | 0.04152 |
|  | Error | 1.210 |  |  |
| Superoxide dismutase (SOD) | Treatment x time | 0.18346 | 5.22 | 0.00003 |
|  | Error | 0.03515 |  |  |
| Peroxidase (POD) | Treatment x time | 0.58800 | 42.98 | 0.0000 |
|  | Error | 0.01368 |  |  |
| Polyphenol oxidase (PPO) | Treatment x time | 0.16532 | 5.51 | 0.00002 |
|  | Error | 0.02998 |  |  |
| Proline | Treatment x time | 17.548 | 31.06 | 0.0000 |
|  | Error | 0.565 |  |  |
| Protease | Treatment x time | 0.25026 | 3.21 | 0.00272 |
|  | Error | 0.07803 |  |  |
| Chlorophyll a | Treatment x time | 0.03779 | 2.48 | 0.01566 |
|  | Error | 0.01524 |  |  |
| Chlorophyll b | Treatment x time | 0.01210 | 2.14 | 0.03588 |
|  | Error | 0.00566 |  |  |
| Carotenoids | Treatment x time | 0.25043 | 4.74 | 0.0001 |
|  | Error | 0.05278 |  |  |
| Chlorophyll a+b | Treatment x time | 0.08550 | 4.08 | 0.0004 |
|  | Error | 0.02094 |  |  |
